# Using the D-DANP-mV Model to Explore the Continuous System Improvement Strategy for Sustainable Development of Creative Communities

**DOI:** 10.3390/ijerph14111309

**Published:** 2017-10-27

**Authors:** Lei Xiong, Cheng-Lein Teng, Bo-Wei Zhu, Gwo-Hshiung Tzeng, Shan-Lin Huang

**Affiliations:** 1 College of Creative Design, Asia University, Lioufeng Rd. 500, Wufeng, Taichung 41354, Taiwan; kmt20005@gmail.com (L.X.); teng@asia.edu.tw (C.-L.T.); 2 Graduate Institute of Urban Planning, College of Public Affairs, National Taipei University, University Rd. 151, San Shia District, New Taipei City 23741, Taiwan; zhubowei301@gmail.com (B.-W.Z.); samlin0668@gmail.com (S.-L.H.); 3 Faculty of Humanities and Arts, Macau University of Science and Technology, Avenida Wai Long, Taipa 999078, Macau, China; 4 Department of Tourism Management, Tourism School, Sanming University, 25, Jingdong Rd., Sanyuan District, Sanming 365004, China

**Keywords:** creative economy, creative community, sustainability, continuous improvement strategies, DEMATEL-based ANP with modified VIKOR model (D-DANP-mV model)

## Abstract

With globalization, the notion of “creative city” has become a core concept of many cities in the world development policies, with real properties being upgraded or used to change, renewal is being conducted, and creative industries are emerging. This trend has reached its peak in the past decade, with different forms and scales gathering global development momentum among the creative communities to promote the development of creative economies. In recent years, however, there was still skepticism about the sustainability of the current creative communities. Many scholars have pointed out that signs of unsustainability have begun to appear in many creative communities. To overcome these obstacles, the development of rational and highly effective improvement strategy requires a dynamic thinking process. Therefore, this study employs the DEMATEL-based ANP with modified VIKOR (D-DANP-mV) model in presenting an assessment framework for the sustainability of creative communities. This system is used to assess the sustainability of current creative communities and determine how to solve their problems. Thus, continuous and systemic improvement strategies can be developed to achieve the aim of sustainable development. Two creative communities in Taiwan, Taichung Cultural and Creative Industries Park (TCCIP), and Shen-Ji New Village (SJNV), are used as case studies in this study. Based on the concept of systematic improvement from fundamental issues, the results indicate that the improvement priorities can be determined by applying the D-DANP-mV model. This approach is different from those found by a conventional method with the hypothesis of independent criteria (e.g., diversification of creative talents in TCCIP), and cannot use for performance improvement (e.g., only can be used for ranking and selection among alternatives). Considering these points, unreasonable premises, biased errors, and lack of some real application functions in the process of resource allocation could be more efficient improvement strategies generated in this proposed model.

## 1. Introduction

Disillusion with the economic policies of the 1990s, mainly in the field of property-led approaches that were overriding cultural policies, has attracted attention to cultural and artistic activities in a new wave of city regeneration [1]. Creative industries can contribute to increasing the attractiveness of a city, resulting in a spillover into the service-oriented economy in general [2]. At present, the cultural and creative industries have become key economic drivers and the basis of a new urban viability orthodoxy. To support the creative industries, governments around the globe are building various forms of creative clusters, and on different scales [3]. In this context, the creative communities are revitalizing the cities to consider the creative/cultural economy strategies in a lively milieu of place-based social relationships that has appeared in many parts of the world. In the term “creative communities,” the word “creative” denotes “a person’s opinions, solutions and advices by a novel and appropriate method”; while “community” refers to “the complex social relations that make up the creative locale” [4]. Doyeon and Zhai [5] believe that creative communities are created by individuals of different creative classes in specific locations and/or aggregated areas and that these communities have their own unique appeal and constituent elements. Creative communities should closely link the arts, culture and related industries to form a driving force behind the development of the technical economy in the post-industrial era in order to spur rapid urban development. Yet in recent years, more and more scholars have questioned the creative communities’ current sustainable development prospects [3,6,7]. On the one hand, scholars are of the view that many creative communities are placing too much emphasis on the pursuit of realizing economic goals [1,4,8]. For example, Joubert [1] pointed out that extensive practical experience has shown that a great amount of financial support allocated for the development of creative communities has been consistently utilized for capital investments and, consequently, has not yet been utilized to support the community-led revival toward participatory cultural and artistic activities. Under this trend, cultural commercialisation and instrumentalisation tend to produce cultural banalization, thus eroding intrinsic cultural value [4,6]. On the other hand, scholars have criticized many developing countries that are currently implementing creative/cultural economic policies, only through successful experiences and practices from abroad, and even applying them to the revival of their local communities without careful consideration [8]. This could lead to the “corrosion of neighborhoods” or residents’ identity with the loss of the city itself, followed by the development of a sense of isolation, and accelerate depletion of cultural vitality [8,9]. However, notwithstanding the harsh criticism that has emerged within the scientific world, these policies have been largely put in practice in most major cities around the world [6]. This shows the importance of devising a rational and highly effective improvement strategy to ameliorate some of the many developmental crises currently facing creative communities and allowing them to develop sustainably.

The discussions on this issue clearly need to take into account the concept of sustainability. This concept was first introduced by the Brundtland Commission in 1987 in a report titled “Our Common Future,” which explained the concept as follows: “development that meets the needs of the present without compromising the ability of future generations to meet their own needs” [10]. The concept of sustainable development is now considered to be the most fundamental issue concerning human activities for development [11]. Based on the relevant literature, scholars commonly refer to economy, environment, society, and culture as the four main dimensions of sustainability in studying creative cities and culture-led urban regeneration. The key to achieving sustainable development is to ensure the harmonious and symbiotic relationships among the four dimensions, so that the state of balanced development can be maintained [12,13]. Nevertheless, in practice there are dynamic and complicated influential forces acting among these four dimensions [14,15], and disposable resources are often limited. Clearly, effectively resolving the practical issues discovered to help the creative communities for achieving sustainable development in a timely fashion, are the implementation of the basic ideas to consider before [16]. Therefore, the sustainability of creative communities should not be examined solely from the perspective of a single dimension but must from the combined perspectives of systemic as well as mutual influences, in order to how prioritize improvement agendas by systematics. Hence, the development of a reasonable and highly effective improvement strategy requires both identifying the main practical issue within the current situation and finding the root cause-effect of this issue based on the mutual influences among the assessment dimensions. It is only through exploring a continuous and systemic improvement strategy that the issue could be resolved at its inception and improvements could be implemented, thereby helping creative communities achieve the aspiration level [17,18].

Thus, this study examines, evaluates, and improves the key influencing factors that affect the interrelationships of sustainable creative communities toward reaching the aspiration level. Based on this, the means to make systematic and continuous improvement strategies for creative communities can be explored to realize the aspirations for achieving sustainable development. First, based on literature review, the criteria (attributes or factors) for promoting the sustainable development of a creative community are extracted from the perspectives of the four dimensions of sustainability. On this basis, a systematic approach was established to assess sustainability of the creative community indicator framework, and then pretests of indicators were used to determine the key evaluation criteria of creative communities. Second, the DEMATEL-based ANP with modified VIKOR (D-DANP-mV) model was used to evaluate contemporary creative communities. The D-DANP-mV model is a hybrid technique that combines three techniques. The DEMATEL technique was applied to obtain an influential network relationship map (INRM) for proposing some systematic observations and the influential weights (IWs) of the evaluation criteria according to a process referred to as the DEMATEL-based analytic network process (ANP) or called “DANP” for realizing priority criteria. Thereafter, a modified VIKOR method was applied to evaluate two creative communities in southern and western districts of Taichung with creative workers in empirical cases for discovering problems. Then the systematic and continuous improvement strategies were developed in the real world case study. As many larger Asian cities have seen labor-intensive manufacturing relocate to other cities or other countries, their administrations have also turned to cultural economy and creative city strategies for revitalization [9]. Among these cities is the second largest city in Taiwan, Taichung city, which is moving to become a creative city with important urban development, cultural facilities, and amenities under construction [16]. During this process of development, as recipients of strong support and promotion from the Taichung municipal government, the Taichung Cultural and Creative Industrial Park (TCCIP), located in the southern part of Taichung City, and Shen Ji New Village (SJNV), located in the western part of Taichung City, have already developed into two of Taichung City’s most important cultural sightseeing destinations. Additionally, these two creative communities have already become well-known examples of urban idle space activation in Taiwan. However, TCCIP and SJNV also have their own unique practical problems and developmental crises. Although these two creative communities are in different forms and have distinctive features, they are both brimming with vitality and share a common objective: pursuit of sustainable development. Therefore, the author has chosen these two Taiwanese creative communities as case studies in this research.

The rest of paper is organized as follows: Section 2 reviews previous studies on creative communities, the four main dimensions of sustainability, and the positive influence factors, thereby determining the evaluation criteria for creative communities. In addition, the pretest of indicators, superiority of the D-DANP-mV model, and the research procedure are presented. Section 3 describes the empirical case selection, data collection, analysis, and results, followed by a discussion and implication. The final section focuses on the crucial findings and puts forward the future research directions.

## 2. Evaluation Framework, Research Methods and Procedures

### 2.1. The Four Dimensions of Sustainable Creative Communities

In previous studies, scholars generally considered the economy, the environment, and the society as the three main dimensions of sustainability [11,19]. As mentioned above, the most significant reason for the emergence of creative communities was promoting the country’s creative economic development, creating a new urban image, and thereby making the city more attractive to mobile capital and mobile professional workers [4,7]. Therefore, similar to many sustainability studies in other fields, the issues related to economic dimensions of creative residence or creative/cultural space prioritized by scholars and so studied more thoroughly and deeply [14]. Nevertheless, since the beginning of the twenty-first century, with the accelerated processes of urbanization and metropolitan, the public is increasingly concerned about the serious global environmental problems more and more. Accordingly, many of the issues related to the sustainability of urban construction, urban regeneration, or sustainable community building have gradually shifted research priorities from overwhelming economic dimensions to discussing the human/community–nature relationship within various contexts, landscapes, and ecosystems in order to seek a balance [11,15].

In recent years, some researchers have proposed to examine how some of the actual creative communities developed. This could identify (empirically) and explain (conceptually) the failure of these actual creative communities to engage with social justice and social inclusion instead of proposing ideal strategies to achieve economic and environment goals [1,20,21]. Clearly, the social dimension raised here was to complement and perfect the evaluation of sustainable creative communities, which had been seriously neglected in previous studies. In addition, there has been an increasing number of academic interests in considering culture as another important aspect of sustainable development [15]. This emphasizes the importance of the cultural dimension to sustainability and the key role that art and culture play in social change [12]. In previous studies, scholars aimed to integrate the idea of culture as a way of life into the entire system of planning, arguing that cultural industries, craft, local heritage, and other “cultural resources” functioned as a catalyst for development [9,22,23]. Therefore, the following section is divided into four parts, dimensions of the economy, environment, social, culture. The key influential factors within the four dimensions, which have a large influence on the sustainable development of creative communities, are each discussed in detail.

#### 2.1.1. Economic Sustainability

In the present study, the economic sustainability of creative communities refers to the increase of its overall economic vitality and competitiveness, the stable growth of economic income, as well as the best coexistence and coordinated development of the economic sector in the creative economy [14,24]. To address the challenges faced by creative industries in the era of globalization, scholars have emphasized the importance of fully utilizing advantageous local resources [24]. This was in order to increase the specificity of economic activities and ensure that small regional differences play a positive role in economic activities and thereby assist creative industries in obtaining distinct competitive advantage under the effects of economic globalization [25,26,27]. For example, the attributes which may allow a locality to attract increasingly mobile capital clearly may include the “traditional” factors of lower costs, proximity to infrastructure, size and nature of local markets, availability of expertise, and others [24]. In this case, Doyeon and Zhai [5] also suggest that if we want to ensure that creative communities will enjoy continued, stable, and prosperous growth, we should first work to improve communities’ ability to attract various types of creative people as well as the broader public. This is because promoting the development of creative industries requires sufficient human resources with specialized skills to serve as the main driving force of the sustainable development of the creative economy. Diversified human resources not only deploy different skills, aptitudes, and sensibilities, but also provide the possibility for boosting innovation and creativity. The exchange and interaction between creative workers can improve the spillover effects of information, enhance the understanding of cultural differences, and contribute to the new creative processes [14,27,28].

Moreover, in the production of all kinds of goods and services, the symbolic dimension has gained central importance, which has led to an almost universal aestheticization of goods and everyday practices thanks in particular to publicity and design [6]. For creative community, the overall increase in symbolic value is the key factor in attracting followers, e.g., tourists, product clients, investors, and new members [12]. Under the model of a creative city, the increase in symbolic value arises from the full utilization of tangible and intangible cultural capital, high-level and authentic cultural capital is the real reason why the ability of creative communities continue to attract new mobile capital [29,30]. On the other hand, as the continuous competition between similar projects increases every day and the product life cycle continuously accelerates, creative communities should put more emphasis on producing creative products [31]. Everything, from community created content to enterprise output (products and services) must continuously update and improve in order to maintain a core competitive advantage [32,33]. Jang et al. [34] suggested that regional and district policy makers should consider product innovation policies based on each sub-cluster’s specific product innovation potential, due to the heterogeneous agglomeration effects in product innovation activities. In addition to the above mentioned, creative communities must establish a stable financial foundation to secure the allocation of basic operating expenditures and facilitate the promotion of cultural and art activities [31,32]. For instance, taxation systems can be established in the city to generate funds for organizing cultural and art activities, investing in the construction of specific cultural and art facilities, acquiring public art works, or supporting artists involved in creating public art [22,35]. In the past few years, scholars have indicated that, during the creative space construction process, we should focus on establishing cross-domain partnerships and promoting creative cooperation that will be more sustainable than in the past due to the public fundraising model [23,36]. This will allow us to further increase the stability of the financial base.

#### 2.1.2. Environmental Sustainability

Many previous studies have emphasized that culture-led urban renewal, a healthy living environment that can meet the needs of the economy and society (including politics) is also important in creative/cultural spaces [5,9]. Furthermore, achieving the balance between the realization of human resource development and the protection of environmental vitality should be the goal of building a healthy living environment of creative/cultural spaces [9,11]. Recent extreme weather events and global trends in climate change have made researchers take into account the negative effects arising from urbanization. Therefore, creative communities should be implemented with approaches that promote energy conservation and carbon reduction to achieve [37,38,39]. This implies increasing the efficiency of an urban community throughout the life cycle, substituting or reducing carbon-based energy, technological innovation and behavioral changes. Furthermore, renewable resources should be at rate of consumption lower than the rate of regeneration [37,39,40]. Meanwhile, designers should promote the three R’s: re-duce, re-use, and re-cycle, but should also design for up-cycling, which is the conversion of waste and old materials into new materials or products of better quality or a higher environmental value [41,42]. Thereby, the waste generated from a process or component of these systems can become food or raw materials for another process or component, and the waste of reduced resources is almost zero. This approach can develop buildings, communities, and systems that support human and environmen1tal health [41]. Additionally, scholars’ studies of real-life cases have led them to recommend that we should work to ensure that the socioecological environments and natural scenery are not damaged during the establishment of creative communities and places [5,9,35]. They also suggest that sufficient green space should be left during community planning to protect the existing vegetation and aquatic environment and those natural landscapes [5,9]. The roads and the waterfront space should be designed to match the natural scenery [5].

On the other hand, the creative community must create an environment that can nurture a creative atmosphere, inspire creative workers and provide comfortable working and living spaces. That is how we will truly be able to further stabilize creative groups and continually attract new creative talent and community members [5,9]. For creative communities, the quality of community-built environments can be improved through specific strategies, such as the development of urban brown areas, the construction of public open spaces and infrastructure services and the renovation and reuse of idle buildings [27], these strategies not only generate environmental benefits, but also bring new residents and increase consumption of new urban spaces [43]. From another perspective, a sense of identity with the unique and memorable place is part of the basic human yearning for home and connectedness [42]. Buildings and landscapes with distinctive features can enhance their identity and the authenticity of the project environment. Although the form and feature of buildings and landscapes can be imitated, they can never be fully replicated and replaced [24,27]. The conservation and sustenance of these complex urban ecological environments create a unique feature of a place and allow it to develop distinctive cultural capital and individualized creative environments that attract creative individual and encourage the construction of highly attractive cultural facilities. Such positive influences help to build a creative momentum for developing creative communities [27,29].

#### 2.1.3. Social Sustainability

In general, social sustainability suggests a healthy social interaction, protection of vulnerable persons, and respect for social diversity [9]. These values can be enhanced through developing citizenship, expanding access, acknowledging multiculturalism, and developing partnerships or community involvement [1]. First, social inclusion policies must first allow and encourage all members in a region to actively participate in economic, social, and cultural activities [21,29]. Therefore, local governments should provide a wealth of opportunities for citizens to participate and pay sufficient attention to advice and comments from the community base regarding community development, encouraging the development of creative communities through a bottom-up approach [4,8]. Community members’ attitudes can play an important role in shaping the development of the cultural tourism industry and protecting cultural heritage [44]. They are also crucial to suppressing the excessive commercialization of creative communities [5]. Moreover, quick and effective access to public feedback on the feasibility of new solutions is suitable for evaluating the social influence of these polices on existing communities [5]. Furthermore, cultural planning and the construction of creative spaces should be conducted in a way so that a good social environment is provided for all community members and visitors in order to promote a wider range of interactions and exchanges between people [9]. This will not only break the isolation that divides people from one another, but it will also strengthen people’s awareness of the creative spaces and form cohesiveness between community members [8]. For example, Visanich and Sant [45] recommend that creative/cultural spaces should not be mainly presented as a venue for cultural and artistic exhibitions, but should focus more on becoming an urban space where the masses can actively participate and which facilitates the development of various types of informal interactive activities and creative projects. Moreover, interdisciplinary cooperation between artists and other creative workers can be utilized as a key reform mechanism to promote intercultural cross-pollination among various social networks and urban environments [12,46]. Once again, creating places within creative/cultural spaces where community members can gather together for informal and shared social learning is a new trend in recent years around the world [47]. This can be used to instill the principle of learning within the creative field of all organizations and members of this study, to encourage them to understand and explore the dynamic changes and the historical and cultural contexts of their urban living and working spaces [9,12].

In the area of information technology, convenient and effective digital information technologies can be used to create adequate and novel learning environments [32]. Therefore, the importance of information management and communication in creative communities must be emphasized. Communication and information sharing should be directive, open, and effective. The wider dissemination of community information and the increased accessibility and accuracy of information communication will encourage community consensus and social interaction between the creative workers and community residents to provide a foundation and medium for developing new cultures and innovations [8,32]. On the other hand, several urban regeneration projects worldwide have resulted in a prevailing homeless population, including artists and other creative workers who have been forced to vacate their homes [9,35]. Artists and other creative workers play an important role here since they are sometimes perceived as pioneers of gentrification when they bring their cultural capital to a certain district or region [11]. This phenomenon not only indicates an inability to achieve social sustainability, but also contributes to the loss of a driving force for creative economic development and the spirit of creative communities [14]. Therefore, scholars have suggested that policy-makers and developers can help the middle- and low-income residents of creative communities by providing adequate life quality, ensuring basic work rights, and working to end all forms of discrimination [9,21,35]. They must also address infrastructure issues and affordable housing. Due to the nature of their avocations, they need to think more concretely about equity.

#### 2.1.4. Cultural Sustainability

This study considers cultural sustainability as the ability to create local cultural content and embed indigenous idioms in cultural “products”. These support the creation of unique cultural forms that reinforce a sense of local identity and, indeed, of nationhood, particularly in the face of globalization and the potential of homosexuality [9,35]. Such cultural sustainability should be able to nurture cohesion and develop common identity, without suggesting a xenophobic rejection of external influences. For example, Doyeon and Zhai [5] suggested that the creative community should be an “open” field where the expression of artistic and cultural creativity should not be divided into geographical, racial and professional fields, and policymakers should respect and promote cultural and artistic diversity. Under this view, creative development will benefit if the environment is enriched with cultural expressions that broaden the worldview of the artisan, as well as enabling them to use the services of other professionals who can advise them and increase their productive capacity thanks to the training and broad perception in the creative field [48]. From the perspective of cultural diversity, preserving marginalized ethnic minorities and indigenous people and protecting their conventions and cultural rights in a constantly evolving and changing contemporary society in practice requires emphasizing and promoting the differences between local and global cultures, while also supporting community development through local cultures and conventions [15].

It is noteworthy that cultural and artistic activities of nongovernment organizations, citizen groups, and grassroots public organization exhibit a bottom-up spontaneity that can increase the cultural energy of creative communities, thereby preserving local community culture and ensuring new community developments [5,8,9]. Furthermore, arts and community cultural activities can contribute to community identity, increase community pride, and foster participation [9]. Additionally, systematic collaboration between community members will stimulate their potential, and rich, high-quality cultural and artistic events will display the creative community’s vitality and glamor [5]. This will also greatly enhance the community’s ability to attract creative talent [35]. On the other hand, in both academic and practical fields, there is a growing awareness that cultural heritage is an integral component of social and community wellbeing [9,15]. Whether material or non-material cultural heritage can become the cultural and creative value of a particular area or region and it is thus necessary to protect it and support its growth to appropriate and harness its essence [48]. The protection and conservation of cultural heritage can be regarded as a crucial factor for a society to develop creative communities, establish connections with local and traditional cultures, and encourage a sense of cultural identity among residents members [5,13]. 

### 2.2. Evaluation Framework for Sustainable Creative Communities

**Figure 1:**
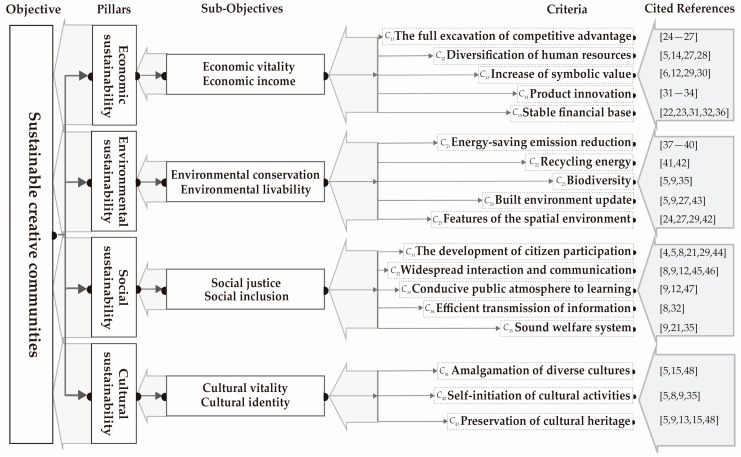
Constructing an evaluation framework for sustainable creative communities.

In establishing the assessment framework, what first needs to be identified is the ultimate objective of this study. Second, the basic aspects for the achievement of this objective also need to be identified. Third, the sub-objectives derived from each aspect need to be organized. Lastly, key criteria are extracted from previous research, through which these sub-objectives may be achieved. In summary, the elements are visually organized into a framework outlining the objective, aspects, sub-objectives and criteria of overall system indicators that can enhance the sustainable development competency of a creative community (Figure 1).

In Figure 1, it should be further explained that since the study aims to establish a comprehensive indicator framework that can be used to assess the sustainability of creative communities, “culture” will be regarded as parallel study with three commonly recognized main aspects under the research objectives [9,15,49]. It will hence be included in the assessment system of this research as the fourth aspect. This is because if the cultural dimension is not given sufficient weight while considering the issues concerning the sustainability of creative or cultural spaces, important underlying issues might be overlooked in research discussions [12,49]. The extraction of all criteria in Figure 1 is based on the discourses in the previous two sections and has been carried out to achieve all the sub-objectives under the four aspects. However, all the criteria extracted in this section have yet to be conclusively confirmed. In the following section, a pre-test is used to determine the key assessment criteria, from which the contents of the final assessment questionnaires can then be confirmed.

### 2.3. Pretest of Criteria

In order to identify the reliability of an evaluation framework, the pre-test in this study is conducted in two stages with a semi-structured questionnaire. The first stage of a survey is implemented with open questions to find out what the experts think about each of the criteria mentioned in the previous section. Which means the experts can modify the dimensions and criteria’s name and descriptions of an evaluation framework by their experience. At the same time, they also can give a suggestion for adding another dimensions or criteria. Experts widely believe that the most significant problem is that the evaluation framework contains some guidelines which are not named precisely enough. These names cannot clearly and accurately express the true meaning of the guidelines and will often mislead readers. While sorting out the pre-test questionnaire, it was discovered that the evaluation framework contained seven guideline names that had to be modified, namely the *C*_11_, *C*_12_, *C*_14_, *C*_21_, *C*_24_, *C*_31_ and *C*_34_ guidelines in Figure 1. The author has edited the names of these guidelines according to expert advice (see Table 1).

After the first stage, the framework that include dimensions and criteria have been defined. The second stage is to test each criterion by using the important level of closed questionnaire. From this, the importance of each criterion can then be determined based on the average index. The experts will be rating each criterion from 0 to 10 according to their own experience and perceptions, with 0 indicating “extremely unimportant” and 10 indicating “extremely important”. If we apply the Likert 5 scale to the scoring scale in this study, 5 stands for “normal” and 7.5 is the semantic cutoff point of importance. Therefore, the study set 7.5 as a threshold. Thus, a value of more than 7.5 indicates an importance of more than 75%. This in turn means that the experts believe that such criteria are important to this study’s evaluation framework and should be retained. However, regarding the criteria that fall between 5 and 7.5, this study will use organized, expert interviews to evaluate what these guidelines are, whether they should be retained and/or how they must be improved. The author organized expert interview materials and then conducted another questionnaire to gain advice from experts on changing the guideline framework. This process continued until all values were less than 7.5.

In the second phase of the pre-test, we discovered that after the changes in the first phase, the evaluation framework did not have any guidelines with scores less than 5, nor were there any scores between 5 and 6. This means that, in the eyes of the experts, these guidelines that had been taken from the crucial documents were all filtered down to the most important ones, after the first stage of the pre-test. However, among the initial 18 criteria, there were seven criteria values between 6 and 7.5, of which four were between 7 and 7.5. These criteria include: continuous innovation of the product (C_14_) (value is 7.000); widespread interaction and communication (C_32_) (value is 7.467); conducive public atmosphere to learning (C_33_) (value is 7.333); high-quality transmission of information (C_34_) (value is 7.067). This means that the first stage of the pre-test did not completely correct the validity of the evaluation framework. Thus, in order to further correct the evaluation framework for this research, more expert interviews were needed during the second phase to discuss the aforementioned guidelines that scored less than 7.5.

The expert interviews in the second stage of the pre-test confirmed that they regarded the four criteria as unsuitable for different forms of creative communities, based on their personal practical experience; therefore, they gave lower ratings to the importance of the four criteria. However, even more experts are of the view that, regardless of the types of creative communities, these criteria are the key factors that help creative communities achieve sustainable development. Many previous studies have pointed out that many of the failed cases have resulted in non-sustainability because they neglected all or some of the five criteria. Experts emphasize that in different types of creative communities, these four criteria can be expressed in different manners, and that these criteria have important influence. During the interviews, this view was also echoed by experts who held different views in the first stage of the pre-test. Therefore, the four criteria, whose values were between 7 and 7.5 should are maintained in the assessment framework of this study, and they require no amendments.

In addition, there were also three criteria values falling between 6 and 7: diversification of creative talents (*C*_12_, value is 6.200), energy conservation and emission reduction in buildings (*C*_21_, value is 6.000), and a sound welfare system (*C*_35_, value is 6.733). The expert interviews reveal that three criterion values were lower than 7.5 because the three criteria were not completely defined in the survey, so the experts were unable to make an effective judgment on their importance in the first stage. First, the experts suggest that the description for the diversification of creative talents (*C*_12_) should focus on the broad-ranging nature of the need for human resources in the cultural employment systems, and not only on the concentration of a certain human resources. Second, the description for the energy conservation and emission reduction in buildings (*C*_21_), the experts consider that it is inadequate for addressing environmental issues that will become increasingly severe if they merely emphasize more efficient use of materials and energy. Therefore, the experts propose that the description for this criterion should emphasize advocating the development and utilization of renewable energy in the creative industries, as well as establishing environmental awareness in society using publicity and cultural events. Third, the experts suggest that the description for the criterion concerning the sound welfare system (*C*_35_) in the pre-test should highlight the need for care and support for low-income creative workers. As demonstrated above, after amending the descriptions of the criteria in accordance with the experts’ opinions, another round of survey was conducted with the experts to determine the importance of the three criteria. The results indicated that the values of these criteria that had been amended have become higher than 7.5, which signifies that all the present criteria have been confirmed as important and effective. After the conclusion of the pre-test stage, all the criteria names in this indicator system and their detailed descriptions are set out in Table 1.

### 2.4. Evaluation Methods for Sustainable Creative Communities

In previous study, scholars typically adopted a qualitative methodology to examine the issue of non-sustainability in the development of creative communities. The qualitative data of these studies were mainly collected through methodologies such as field investigations, interviews and observations, and the data were analyzed with inductive reasoning [3,4,7,8]. When focusing on smaller and more concentrated samples, using a qualitative approach helps to achieve a more thorough understanding of the specific cases. This allows for a deeper understanding of causal connections in the study issue as well as a more nuanced and comprehensive discussion that includes diverse viewpoints. Moreover, compared to quantitative research, the results of qualitative research are more susceptible to influence of the researchers’ understanding and proficiency of the methodology [50]. Apart from that, the previous literature shows that, from the perspective of sustainable development, scholars increasingly use qualitative analysis to understand and assess the current developmental situation of creative communities. But they seldom achieve a systematic assessment and are, moreover, unable to generate a concrete improvement strategy based on the assessment results.

To date, sustainability science still seeks to integrate quantitative sustainability ideas into a new definition of how sustainability can be achieved [19]. As with most assessment research using quantitative analysis, what first needs to be done in the course of research on sustainability, is to analyze the assessment tools. In order to understand more clearly the priority of all criteria in the assessment framework, the weight of each criterion must be determined. The common method used for solving such problems in previous research was the analytic hierarchy process (AHP) [51,52], however, this method involves the problematic assumption that the criteria are all independent. But in practice there is always a certain degree of correlation or influential interrelationship among different factors, and even a certain degree of conflict between multiple attributes [53]. Therefore, scholars began to use the analytic network process (ANP) instead of AHP for assessment research [54,55,56]. Earlier, Saaty [57] proposed the ANP to solve dependence and feedback problems between dimensions (clusters), criteria (inner dimensions/clusters) in diagonal matrixes until they are assumed to be independent (zero matrix) or assumed to be self-related (identity matrix, I), and weighted supermatrixes obtained using equal weights (assumptions). As such, using ANP to determine the assessment of choice in practice is likely to result in relationships between the dimensions and criteria where their mutual effects are unclarified. This will affect the accuracy and applicability of the results of the analysis of decision-making. However, real-life problems are often quite complex because there are many mutual influential relations among the items. The D-DANP-mV (DEMATEL-based ANP with modified VIKOR) model is a hybrid method in the Multiple Criteria Decision-Making (MCDM) field. Based on the systematic concept, the main contribution of this model is to provide an improvement strategy for decision makers (DMs) and thereby improve performance of the alternative. The model breaks down the assumption that the relationships among variables are independent. It also emphasizes the influential relationships among the variables that replace the correlative relations in conventional regression analysis. From this perspective, the D-DANP-mV model is especially suitable for solving complex and practical problems.

### 2.5. Purpose, Target and Process of the Research Method

The D-DANP-mV model is a hybrid technique that combines three techniques: DEMATEL, DANP and modified VIKOR [58]. Initially, the influential network relationship map (INRM) of the dimensions and criteria are obtained using the DEMATEL technique [59]. Then, in order to determine the influential weights (IWs) of the dimensions and criteria, the DEMATEL-based analytic network technique (DANP) is applied. Finally, the modified VIKOR method is applied to calculate the ratio of the gaps between actual performances and the aspiration levels [60,61]. It should be noted that measuring the aspiration level, instead of the relative standard, is used as a benchmark to avoid “picking the best apple from a barrel of rotten apples.” Finally, we can find the criteria with larger gaps in alternatives, so that improvement strategies could be made through INRM analysis (based on the concept of influential resource) for DM. In summary, by using the D-DANP-mV model, this study explores the means to evaluate the sustainability of a creative community and then make improvement strategies towards achieving a truly sustainable creative community.

**Figure 2:**
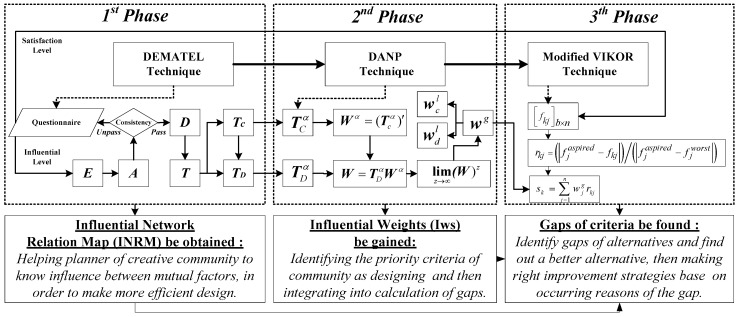
Target and process of the research method.

In order to achieve a lasting creative community, in this paper the D-DANP-mV model is divided into three phases. Each technique corresponds to one of the phases and there are different targets for each phase of the D-DANP-mV model, as shown in Figure 2. D detailed steps are described in Appendix A. For planners of a creative community, the influence relationship of each factor is important information for designing the community. The result of the first phase is an Influential Network Relation Map (INRM) for developing a sustainable creative community, which helps to design a more effective community by systematics. The results of the second phase are also important for planning a creative community, since this phase can obtain the influential weights (IWs) for each criterion to identify community design priorities. The third phase can then find gap’s criteria for each alternative by summarizing the IWs. The planner can rank each alternatives according to the results of this evaluation. Finally, creative community improvement strategies for sustainable development can be obtained by seeking the criteria of larger gaps in alternatives, and then finding what factors can affect these criteria by using INRM.

## 3. Empirical Case Study: Analysis on Influential Degree among Evaluation Index System

### 3.1. Empirical Cases Description

Taichung City, Taiwan is known as a “cultural city”, whose cultural capital has developed high historical and economic value after several generations of accumulation. This cultural capital is the most important foundation for creating a “creative city” and shaping urban characteristics. Accordingly, the Taichung city government is actively promoting the development of creative industries and the construction of related infrastructure. The introduction of this series of new policies means that Taichung City will progress from the concept of a “cultural city” toward the practice of a “creative city”. Under this development trend, the Taichung Municipal Government initiated two construction plans for creative communities in the southern and western districts of the city center and continues to give policy support. These policies seek to create an activation and reuse project of underused urban space with certain symbolic and exemplary functions. One of these two is the Taichung Cultural and Creative Industry Park (TCCIP) in the Southern District of Taichung City, and the other is the Shen Ji New Village (SJNV) in the Western District. In this study, two creative communities are used as case studies to assess their sustainability, and some suggestions for improvement are provided based on the findings.

First, the predecessor of both cases is an underused urban area with a large number of historical buildings. Of these two, the predecessor of TCCIP is the Taichung winery, and its creative community construction program launched in 2009, and SJNV in 2015 began to set up provincial government dormitory. In addition, TCCIP is only one kilometer from Taichung Railway Station, and is also located near both Taichung City's famous traditional market and a beautiful and well-equipped city park. In contrast, the SJNV is located in a dense residential area of the city, surrounded by creative/cultural spaces that have developed in recent years. After the start-up of the creative community construction program, the traditional buildings in these two cases were completely preserved and restored, while the infrastructure construction and green landscape project were implemented in public open space.

The main differences between the two cases are as follows. The TCCIP is much larger and covers an area of about 5.6 hectares with buildings on the site having their own characteristics. In addition, there are many public works of art on public open space, which are designed to interpret and express the old community culture, many of which are created by artists working in legacy facilities and structures of the old wineries. The SJNV covers an area of only 0.52 hectares, of which the environmental characteristic is reflected by the site within the whole building community. The public works of art by creative workers in this project are placed on the facade of the building, while walking lines and public open space express the workers’ concepts and attitudes.

Second, most of the construction space in the TCCIP is planned as an exhibition site, whose three buildings also provide training courses for cultural and artistic education institutions. In the creative community only a “Cultural and Creative Development Center” can provide creative workers places to set up personal studios or small companies, but the center is not currently open to the public, and interaction and communication between the people and creative workers mainly occurs in exhibitions and during culture and art education. The architectural space within the SJNV is mainly planned according to two types. One of these is called the “Pick Stars Plan” unit, which is intended to provide an entrepreneurial base for youth creative workers. The other is used by the professional management team responsible for investment and operation, and is planned to be established at the cultural and creative stores. The surrounding residents and visitors can conveniently access all the cultural and creative stores at will and interact extensively with the creative workers, but the “Pick Stars Plan” unit within the creative studio has not been yet open to all visitors. At present, the TCCIP is fully operated and managed by the Taiwan Ministry of Culture and the cultural property administration, whereas the operation team of SJNV is composed of Taichung City municipal government together with professional companies and non-profit non-governmental organizations. The two creative community operators have developed a corresponding support program for low-income workers so that people receiving benefits in the TCCIP enjoy certain preferential policies for leases and uses of each site. Site rents in the SJNV are slightly higher than in the TCCIP, but young creative workers in the “Pick Stars Plan” unit receive monthly grants from the Taichung City Government. According to the current attendance rate, only a handful of creative workers have chosen to enter the TCCIP for a long term, and many open leasing sites are still vacant. Most individuals choose to rent a site in the creative community only for short exhibitions. In the SJNV, after two years of rapid development both in the “Pick Stars Plan” unit and on the cultural and creative stores located on the first floor have all been occupied, covering many fields of art and design.

Third, TCCIP and SJNV have completely preserved the architectural heritage of their sites. In addition, visitors to the TCCIP can also learn about Taiwan’s traditional winemaking and wine culture by visiting pavilions and participating in related cultural and artistic activities. However, all cultural and artistic activities in the creative community must be approved in advance through the network after submitting relevant data to the management. Only after the adoption of a standardized audit can activities be held in the designated venue as scheduled. In addition, there are many venues in the creative community planned for use by training classes. Currently, many foreign cultural training institutions are actively using these venues as auditoriums for cultural and artistic education. A brief description of these cultural and artistic activities is promoted through the official TCCIP website and by traditional media. Similarly, in the SJNV visitors can also experience many traces of life in the provincial government staff quarters. Furthermore, there is a “little snail” creative market event scheduled for the third Saturday of each month, which promotes SJNV tourist attractions and the gradual formation of new community characteristics. At the same time, any organization’s cultural and artistic activities can all be held here through a simple and convenient application process. However, the creative community that carries out the dissemination and management of information mainly uses traditional media, so it is not currently equipped with digital public information facilities and has not established an online public home page. Generally speaking, by now these two cases have become important cultural tourism destinations in Taichung City. But for artists and other creative workers, the TCCIP is more like a stage that gathers design and art exhibitions, whereas the SJNV is more like a "creative incubator" that provides the base for training and promoting its young creative workers.

### 3.2. Data Collection

This section introduces the data collection and interviewee selection process. In this research, the first and second phase (the DEMATEL technique and DANP technique)’s questionnaires were distributed to 18 experts. The third phase’s (Modified VIKOR technique) questionnaire was distributed to 92 experts. In keeping with the purpose of this research, the majority of these experts came from two different groups. The first and second phase of this research sought to understand how we can spur sustainable development of creative communities. Therefore, the recipients of the D-DANP questionnaire had to be experts and/or professionals who had either researched theories related to or actually conducted planning for creative communities, management, etc. Because industry, government and academia represent different types of stakeholders, people from these three fields all had different advice, despite their common objectives. Thus, a relatively strict interview method was required. Therefore, the research interviewed experts from industry, academia, and government, respectively and then organized their advice. The third phase sought to understand the relative performance of these two real-life examples with regards to sustainability. Therefore, the interviewees in this stage had to be experts who had long-term working experience in manufacturing firms in TCCIP and SJNV (creative workers), as their familiarity with these real-life cases and understanding of the topics of this research give them reliable judgment. Next, we will introduce the background and qualifications of the interviewees in more detail as well as the questionnaire distribution and collection methods.

In this study, the D-DANP questionnaire was administered over a period of 3 months, and 18 questionnaires were distributed, then 15 valid and three invalid questionnaires were received. Nine of the fifteen experts were scholars with urban planning and landscaping backgrounds. They all possess titles of Associate Professor or higher and are currently conduct academic research in famous higher education institutions. Even more significant is that they all once led or participated in creative community related survey research and planning. Three of the experts are currently researching how to sustain creative communities. In addition, four of the experts are professional planners who worked in famous planning organizations and have at least 5 years of work experience and Master’s degrees. All of these four experts have participated in site planning, design, construction and landscaping design of creative communities. In addition, they all personally interviewed people in actual East Asian creative communities before. The last two experts are local government administrators. They have ten years or more work experience and doctorate degrees. One of them once served as one of the Taichung Cultural and Creative Industrial Park’s (TCCIP) main decision-makers. The other one was an important promoter of the development of TCCIP. The former expert has published over 10 theses relating to the protection of cultural artifacts. In the last 5 years, the latter expert has been working to spur the development of creative communities in Taichung’s surrounding cities and counties. Their unique, rich, personal experience gives them insight on how to spur the sustainable development of creative communities. The 15 experts began to be instructed on the research questions and objectives, how to respond, and completed the questionnaire after the note questionnaire. To improve validity, questionnaires were administered in the form of structured interviews, during which any confusion about the questions was clarified by the authors. The average time for questionnaire completion was approximately 2.5 h.

The third phase, the m-VIKOR questionnaire collected the presentation values for the two case communities in each criterion. In the course of the survey, respondents were asked to evaluate their satisfaction with the 18 criteria in the evaluation framework, based on their own perception of the case community. The purpose of this phase’s questionnaire is to understand the current state of TCCIP and SJNV’s performance in terms of sustainability. Therefore, the most crucial factor was the interviewees had to have a deep understanding of TCCIP and/or SJNV. Since the manufacturers (creative workers) who are based in these two creative communities are the main target of creative communities, they should have a relatively deep understanding and familiarity towards the creative community they are in. Therefore, in order to examine the sustainability of these creative communities, collecting the attitudes and feelings of these two manufacturers (creative workers) with long-term experience in the two creative communities was very important. That is why they were chosen as the subjects of this research. The members of the research team, over the course of three months, interviewed the vast majority of the manufacturers in the TCCIP and the SJNV in order to obtain as many completed questionnaires as possible. For those individual offices that hadn’t yet opened to the public, the researchers arranged private times to distribute the questionnaires. In the end, they distributed 92 m-VIKOR questionnaires, including 11 invalid questionnaires. When interviewees selected the same answer for different questions and/or failed to answer some important parts of the questionnaire, the questionnaire was deemed invalid. Among the 81 valid questionnaires, 36 were related to TCCIP and 45 were related to SJNV. These 81 experts came from various backgrounds, including arts planning, performance arts, craftsmanship, digital technology and many other creative fields. They all had lived in TCCIP and SJNV for at least two years, and the majority of the creative workers were still in the early stages of their careers. Most of them paid close, careful attention to growth trends in their creative community, as said growth directly affected their careers. 23 of the creative worker respondents had already worked in their industry for 10 years and had experience living and working in other creative communities. 87 of them were manufacturers currently stationed in TCCIP and SJNV. 75 of the respondents agreed to be interviewed after they filled out the questionnaire, or 86.2% of them. It was more or less a completely comprehensive investigation.

### 3.3. Results and Discussion

**Figure 3:**
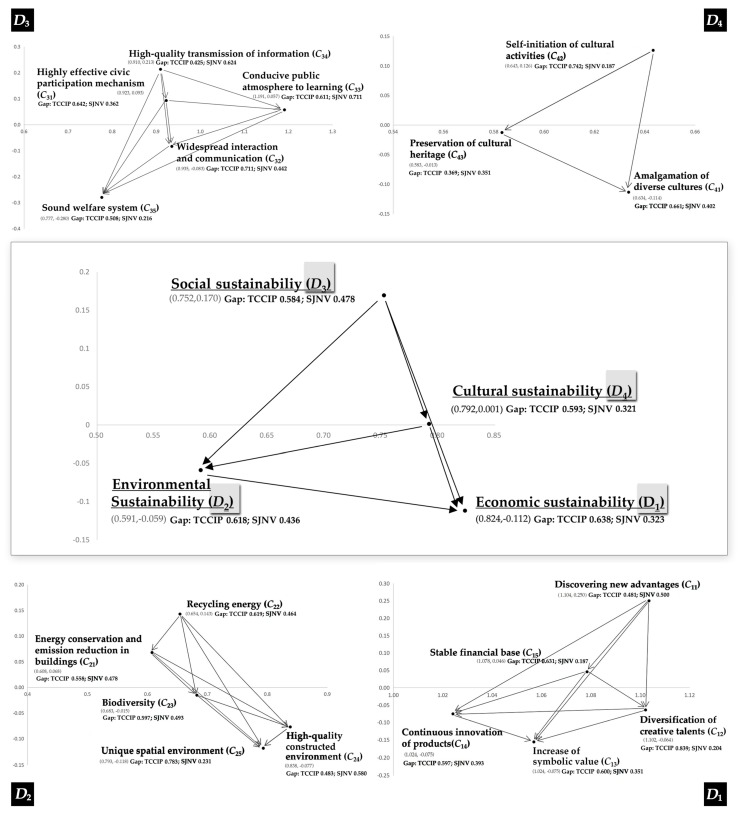
The INRM (influential network relation map) of total influence relationships.

This research applied the DEMATEL technique to analyze relationships among the criteria. With these results, the INRM in Figure 3 clearly expresses the systematic structure of interactions in the improvement model. First, the network relation could be seen as influencing each dimension. INRM indicates the priority of influence as: *D*_3__*D*_4__*D*_2__*D*_1_. These results are interpreted to indicate that, when considering the improvement, the experts all agree that the first priority for improvement should be the aspect of social sustainability (*D*_3_), which can have influence the remaining dimensions of cultural sustainability (*D*_4_), environmental sustainability (*D*_2_), and economic sustainability (*D*_1_). This demonstrates that, even though promoting the creative economy is the initial motive and ultimate goal of establishing creative communities [4], the limited pursuit of economic benefits is evidently not an effective development strategy. To truly realize the sustainable development of a creative community, social, cultural and environmental aspects must be considered because of their powerful influence.

Similarly, from the criteria perspective of INRM, discovering new advantages (*C*_11_) was the most influential criterion in economic sustainability (*D*_1_), and the remaining criteria could be ordered as *C*_15_, *C*_14_, *C*_12_, and *C*_13_. In environmental sustainability (*D*_2_), recycling energy (*C*_22_) was the most influential criterion, and the remaining criteria could be ordered as *C*_21_, *C*_23_, *C*_24_, and *C*_25_. High-quality constructed environment (*C*_34_) was the most influential criterion in social sustainability (*D*_3_); the remaining criteria could be ordered as *C*_31_, *C*_33_, *C*_32_, and *C*_35_. Self-initiation of cultural activities (*C*_42_) was the most influential criterion in cultural sustainability (*D*_4_); the remaining criteria could be ordered as *C*_43_, and *C*_41_.

Based on the INRM (Figure 3) and performance evaluation results (Table 2), improvement priorities are then considered. The improvement strategies of these two creative communities can be established through the results of gap values by using the modified VIKOR technique, based on the IWs found through DANP (Table 2).

Here, the gap value represents the distance between a criterion’s actual performance and its aspiration level, and the total gap value represents the sum of all criterion gap values, which can be obtained from the product of the local weight and gap value of each criterion. From the perspective of total gap value, the SJNV (0.3784) performed better than the TCCIP (0.6110). However, by using the aspiration level (0) as the benchmark, it could be said that both of them have serious problems and several improvements need to be implemented to achieve the overall goal of sustainability. On the level of dimensions, *D*_1_ has largest value (0.6376) in TCCIP and *D*_3_ has largest value (0.4784) in SJNV. This result indicates that these two communities’ development is still in the initial stage, their performance is relatively low, and their weak points may be exposed in certain aspects. In order to develop strategies to remedy these aspects, an in-depth discussion on influencing relationships among criteria is needed, and the following three criteria for the TCCIP exhibited large gap values: diversification of creative talents (*C*_12_) (0.8389), unique spatial environment (*C*_25_) (0.7833), self-initiation of cultural activities (*C*_43_) (0.7417). With the SJNV, the main criterion with the larger gap value is conducive public atmosphere to learning (*C*_33_) (0.7111). The following criteria exhibited smaller gap values: high-quality transmission of information (*C*_34_) (0.6244), high-quality constructed environment (*C*_24_) (0.5800), though their absolute values were still large (compared to 0).

In the traditional sense of improvement, the two creative communities can prioritize the needed improvements by starting from the criteria with the largest gap value. There seems to be a difference that in this model we place greater emphasis on the key criteria which have powerful influences. Nevertheless, by using the influence relationships and the degree of influence for the criteria, detailed discussions of making improvement strategies of two case are operated as follows.

#### 3.3.1. Taichung Cultural and Creative Industry Park (TCCIP)

For TCCIP, on the basis of the performance assessment results, combined with the INRM findings mentioned earlier, the largest gap value, for economic sustainability (*D*_1_) (gap ratio: 0.6376), is influenced by the other three dimensions. Furthermore, the TCCIP also shows greater gap values in environmental sustainability (*D*_2_) (gap ratio: 0.6176), social sustainability (*D*_3_) (gap ratio: 0.5845), and cultural sustainability (*D*_4_) (gap ratio: 0.5930). Therefore, to increase TCCIP’s performance in *D*_1_, the other three dimensions must be systematically incorporated to resolve the root influence behind the problem and devise a package solution to realize continuous improvement. Only then can the gap between the TCCIP and the ideal standard be narrowed. Entering into a deeper discussion on this basis, the performance assessment shows that the criterion currently having the largest gap value is the diversification of creative talents (*C*_12_) under economic sustainability. At present, TCCIP does has a persistent problem of low occupancy rates, particularly where the concentration of creative talents is not as significant as had been anticipated. To resolve this issue which requires urgent improvement, this study proposes that policy makers pay special attention to the criteria under social sustainability (*D*_3_), cultural sustainability (*D*_4_), and environmental sustainability (*D*_2_). This is because the root influences of pragmatic issues lie in these criteria, and the criteria under the dimensions that have greater gap values should be given special attention. Incorporating the mutual relationships among the criteria when investigating the actual issues facing TCCIP in these criteria will be an initial step in developing an improvement strategy to help TCCIP achieve its ideal standard.

First, from the perspective of environmental sustainability, TCCIP has the lowest performance in the unique spatial environment (*C*_25_) (gap ratio: 0.7833) criterion. Presently, many industrial heritage buildings in TCCIP have been properly preserved, and every building exhibits unique characteristics in form and decoration after having undergone repair and restoration. In addition, TCCIP’s many small courtyards are distinctively landscaped. However, such a high gap value shows that the experts commonly do not approve of TCCIP’s performance in this criterion. During the interviews, many experts pointed out that TCCIP is too much like a pure tourist destination, and there is still a large gap between its current situation and its ideal from the perspective of creative workers. Currently, most industrial heritage buildings in the creative community have been transformed into exhibition spaces for cultural and arts exhibition and performance activities. The work and living spaces available for creative workers to lease for long term are mainly concentrated in the 4 transformed factory spaces deep in the midst of the park. The internal space of the buildings is partitioned like company office spaces, after undergoing uniform renovations and shows very little of the historical traces and the building characteristics unique to the creative community. Moreover, the management restricts the creative workers from making unauthorized alterations to the leased premises, where they may only carry out small-scale renovations after approval has been obtained from the management. The results of the research indicate that regardless of how affordable the rent is and how comprehensive the spatial functions are, a rigidly transformed spatial environment, coupled with a commercialized management system, will not be able to attract creative workers to live and work there over the long term.

In order to improve on this situation, this study proposes that the spatial environment created in TCCIP should view the creative workers as its main “target audience”. Many experts highlighted in their interviews that the spatial environment in creative communities should primarily support and serve the development of creative “behaviors” and not merely the display of creative “results.” In managing the TCCIP premises, policy makers should only give guiding principles, provide open-ended direction, and establish spaces where informal social processes are facilitated, avoiding deterministic design [23,45]. Furthermore, new approaches can also be found based on INRM. Under the environmental sustainability (*D*_2_) dimension, the recycling and reuse of resources (*C*_22_), energy conservation and emission reduction in buildings (*C*_21_), and biodiversity (*C*_23_) can all have an effect on the uniqueness of a community’s spatial environment (*D*_25_). This shows that creating natural landscapes with sensitive ecological environments based on environmental protection can help promote the uniqueness of the spatial environment in TCCIP and increase the appeal for creative workers. Similarly, Kong [9] has shown that a natural green landscape with lush vegetation will make the artists feel a sense of excitement and amazement, and they will choose to remain there since such an environment can help stimulate their ideas and creativity. Furthermore, introducing the idea of environmental protection increase the favorable perception of the creative workers for the constructed environment. Fleming [35] pointed out that creative workers will usually participate actively in green building and green design initiatives, presenting their own design proposals or participating presenting art and designs.

Second, from the perspective of cultural sustainability, the crucial issue of TCCIP is manifested mainly in the two criteria of self-initiation of cultural activities (gap ratio: 0.7417) and amalgamation of diverse cultures (*C*_41_) (gap ratio: 0.6611). In Figure 3, self-initiation of cultural activities (*C*_42_) is the most influential criterion under the cultural sustainability (*D*_4_) dimension. In other words, if TCCIP’s current performance in the criterion is improved it will have a powerful and positive effect on performances in the other criteria. In addition, such a change will also trigger a positive chain reaction that will increase TCCIP’s overall performance in the economic and environmental dimensions. At present, the Bureau of Cultural Heritage under the Ministry of Culture is in strict control of all the standard protocols for applications and premises used. The application materials for every activity must be submitted within the stipulated timeframe and the subsequent review process takes nearly a month. With regard to cultural activities that have been approved, the Bureau is only in charge of carrying out follow-up management and supervising and provides almost no assistance whatsoever. Currently, most cultural activities that are approved are organized by universities and colleges in Taichung as well as other cultural organizations and only seldom by nonprofit civic organizations. Many of the cultural activities also take place around the same time every year. What is even more serious is that the lengthy application procedures and complicated regulations in TCCIP dampen the passion and creative ideas of the artists and other creative workers. Also, the large-scale and high-capital cultural activities currently found in this creative community have also caused the small-scale creative workers to feel distanced. The cultural activities carried out annually in TCCIP show signs of homogenization, alienate the creative workers.

Clearly, for TCCIP, the application procedures for activities are in dire need of simplification and modification. Furthermore, the administrators should also strive to help the applicants devise more practical and feasible activity plans through more effective communication approaches, and not merely focus on reviewing the content and form of cultural activities. Incorporating the relationship of mutual influences provided in INRM, this study shows that only the 5 criteria under the social sustainability *(D*_3_) dimension can have an impact on the criterion of self-initiation of cultural activities (*C*_42_). This signifies that promoting social sustainability in TCCIP can fundamentally improve the crucial issue of lack of self-initiation of cultural activities in this creative community. Greater social fairness and inclusivity in the creative community can substantially increase the self-confidence and motivation of nonprofit civic organizations in organizing cultural activities, and a cohesive social consensus is necessary for self-initiated cultural activities to thrive. This increases the possibilities for developing cultural activities and attracts members of the public to participate.

Once again, under the social sustainability (*D*_3_) dimension, the criterion in which TCCIP scores the lowest is widespread interaction and communication (*C*_32_) (gap ratio: 0.7111). Not all of the building spaces in TCCIP are open to the visiting public, and many of the spaces provided for the creative workers’ creative work, exhibition, performances, and sales are closed off. On-site investigations reveal that the visiting public entering the creative community is rarely able to actually have contact with creative workers, and they are more likely to engage only in brief interactions and communication with the staff personnel for the exhibition and performance activities. These interactions were mostly just about the content of the exhibition and explanations. Such interactions and communication can only be seen as superficial, with little actual contribution to forming mutual understanding between the public and creative workers. In TCCIP, even such minimal interactions will disappear at times when there are fewer cultural activities.

From the perspective of the creative workers, external interruptions when they are at work are extremely disruptive, but this does not mean that the workers reject any interaction and communication with the visiting public. Many interviewees have pointed out that they are also very willing to engage with the public at appropriate times and places because inspiration and creative ideas are sometimes derived from views of the public. Based on the relationship of mutual influences between the criteria under the social sustainability dimension in Figure 3, this study reveals that the three criteria of high-quality transmission of information (C_34_), highly effective civic participation mechanism (C_31_), and conducive public atmosphere to learning (C_33_) can have an effect on widespread interaction and communication (C_32_). Nevertheless, TCCIP’s current ratings for C_31_ and C_33_ are lower than that for C_34_, and their gap values are higher than 0.6. It can be observed that promoting the development of civic participation and creating a public atmosphere conducive for learning are the keys to the many real issues faced by TCCIP. Therefore, based on the current state of development, TCCIP requires an effective channel of communication, through which creative workers and other members of the public may voice their opinions, where more members of the community can participate in decision-making for community matters. What is more crucial is to ensure that those cultural strategies coming from the grassroots receive sufficient attention and are really implemented. This is not only to obtain feedback from the grassroots quickly and effectively but also to enable those creative workers from different professional fields to engage in substantial discussions about real issues, thereby fostering their interactions and communication. Similarly, much research has also pointed out that the development of civic participation can balance the growth of non-sustainable phenomena in creative communities, such as excessive commercial development and an increasing sense of social detachment [4,7,8]. Furthermore, there are many premises in TCCIP that are designated as training classrooms, which are made available for temporary use by external arts training institutions for informational teaching activities. There is a wide range of such activities, but the content usually lacks in organization and coherence. Most learners attend only once, and there is very little opportunity for creative workers in the creative community to participate. Therefore, in order to create a public atmosphere conducive for learning, the existing spatial resources must be utilized more efficiently and reasonably. The suggestions of Jakob [7] can be adopted, where public creative workshops can be organized in creative communities to enable local artists and other creative workers to better impart their crafts and knowledge to the public. It enables the residents living in the area and other visitors to truly blend into the creative community, and creates a public atmosphere conducive for learning from the bottom up. For the creative workers, it is not only an opportunity to earn extra income and share their own creative ideas; it also encourages them to step out of their private space and engage in a more widespread and in-depth manner with the local public, where future partnerships may be developed.

In conclusion, although the research results indicate that the performance of the Taichung Cultural and Creative Industries Park (TCCIP) in the dimension of economic sustainability (*D*_1_) is the poorest, researchers in the past might suggest investing resources in this part of “the problem representation.” However, this study puts forward another line of thought, which is to investigate from an “influence perspective,” and invest the resources in an overall package approach. As INRM (Figure 3) shows, the other three dimensions in the evaluation framework of this study have critical influences on the sustainability of creative communities, and similarly, there also exists a relatively big gap in the performance of TCCIP in these three dimensions. Therefore, this study recommends that the local government and the community management department first invest in the corresponding resources to improve the current site planning of TCCIP and the interior space design of some buildings. This is because in order to promote the diversification of creative talents, this problem of the poorest performance of TCCIP in *D*_1_ (Economic Sustainability) is to be improved. The site planning of TCCIP should not place too much emphasis on creating cultural and arts exhibition and performance sites to the negligence of creating a space that is used to develop creative programs and encourage positive public participation. In addition, the community management department should pay more attention to ecological designs and the protection of the natural environment. This is beneficial to the building of a cultural ambience in which creative workers live, create, and communicate; this will further enhance the attractiveness of the creative community to creative talents and other artistic creation lovers. Just as the views put forward by Doyeon and Zhai [5], the success of a creative community depends on the balance between the two forces of community members and community environment; when the balance is broken or the conditions change, a community loses its characteristics of a creative community. Further, in this overall package improvement approach, the local government and the community management department must also try to enhance the spontaneity of the cultural and artistic activities in TCCIP, for the results have clearly shown that there exists a relatively big gap value in the performance of this creative community in terms of such a rather influential criterion. Therefore, this study recommends maintaining the uniqueness and spontaneity of the cultural and artistic activities within TCCIP through the method of increasing the coordination efficiency of the community, with a view to fostering among the community members a surging enthusiasm and motivation to participate and organize themselves spontaneously. Besides the above-mentioned views, based on INRM (Figure 3), it can also be confirmed that the fact that a creative community can truly achieve continuous and stable growth and develop unceasingly depends to a large extent on its own social inclusiveness and fairness and also on whether it possesses great social cohesion as well as healthy and vigorous neighborhood relationships. Thus, on the basis of its present conditions, what urgently needs to improve in TCCIP is the interaction and exchange among people, and so, no effort should be spared to mold a healthier environment of social intercourse. To this, this study still puts forward improvement strategies from the “influence perspective” recommending that the community establishes a perfect civic participation mechanism to enable more community members to participate in the decision-making of public affairs. Besides, this study also recommends that TCCIP makes full use of its existing site resources, creates a conducive public atmosphere for learning, and then proceeds to enable local artists and other creative workers to pass on their artistry and knowledge to those hobbyists more directly and in a long-term and stable manner so as to increase the interaction and exchange between them towards achieving the aspiration level.

#### 3.3.2. Shen-Ji New Village (SJNV)

In the SJNV, even though the gap values shown by the creative community are smaller than the TCCIP’s, there are still areas of improvement for SJNV since there is a fairly large gap in values between the current situation and the ideal standard (Total Gap ratio: 0.3784). It is noteworthy that the performance assessment indicates that the real issues faced by the creative community in achieving sustainable development are mainly in the social sustainability (*D*_3_) dimension. This is significant since social sustainability (*D*_3_) is the most influential dimension in the assessment framework of this study. Therefore, if the real issues discovered thus far cannot be effectively resolved, the sustainability of the creative community will be severely limited, and it will have difficulty developing. Results of the performance assessment clearly show that the criteria with larger gap values are: conducive public atmosphere to learning (*C*_33_) (gap ratio: 0.7111), high-quality transmission of information (*C*_34_) (gap ratio: 0.6244), and high-quality constructed environment (*C*_24_) (gap ratio: 0.5800). Two of the criteria with larger gap values are under the social sustainability (*D*_3_) dimension and are the two most influential criteria under the dimension. Of these two criteria, the one with the largest gap value is conducive public atmosphere to learning (*C*_33_), but the criterion that is more influential is high-quality transmission of information (*C*_34_). Therefore, discussions on improvement strategies for SJNV under the research objective should pay special attention to the issues in *C*_33_ and *C*_34_. Considering the mutual influences between the criteria, a reasonable and effective improvement strategy under limited resources could be developed.

Currently, there is only one class that could help creative workers spread and popularize their professional crafts and engage in relevant educational work: a “hand-painting class” under the “Pick the Stars” program on the second floor of the SJNV. Moreover, this is the only space in the creative community that allows members of the public to truly participate in and experience creative work. During the interviews, many creative workers pointed out that the SJNV spatial accessibility and inclusivity in the creative community is clearly better than in TCCIP. But even though visitors are generally able to freely access most of the premises in the creative community and have many opportunities to engage in interaction and communication with the creative workers there, they are also restricted by several factors, such as the environment, facilities, and time. Thus, members of the public are seldom able to participate directly in artistic creation or learning during their brief visits. But although SJNV has not yet formed a good public atmosphere for learning, the creative community does have the potential to realize this goal, and there are already strong requests from both the creative workers and the public to create a public environment for learning.

Since the *C*_33_ criterion has a huge influence on the assessment framework of this study, improving SJNV’s performance in this criterion will help increase the performance of the creative industry in many criteria. For instance, creating a public atmosphere conducive for learning can help increase the symbolic value and touristic appeal of SJNV. Moreover, in combination with the discussion above, SJNV’s gap value in the widespread interaction and communication (*C*_32_) criterion (gap ratio: 0.4422) will also be reduced. More crucially, the amalgamation of diverse cultures (*C*_41_) criterion, which has reached a gap ratio value of 0.4022 will also be greatly improved due to various factors: the dissemination of knowledge across professional fields, the transmission of local cultural heritage, and the exchange of cultures and arts between different ethnic groups. As such, in order to foster a public atmosphere conducive for learning in SJNV, this study proposes that the local government and administrators, on one hand, direct the creative workers in SJNV into nearby schools and other educational institutions. On the other hand invite students and residents could be invited to participate in artistic creative work in conjunction with cultural events. The results of these activities could be displayed exhibition spaces or other places on the premises for interactive teaching. At present, there is the Xiangshang Junior High School and the Fantasy Story Green Ray Cultural Creative Cluster adjacent to the creative community, it is surrounded by a dense residential area. Both of these are resources for the development of cultural and arts education that could compensate for the lack of space and human resources in SJNV, can providing learners for the long term. In addition, creative workers in the SJNV should not be expected to constantly act as contributors, as they also need to be constantly absorbing new ideas and knowledge in their production and service as well as in their daily lives. The local government and administrators should introduce relevant policies to help the creative workers participate in various educational and interactive activities. However, the preliminary improvement plans mentioned above require effective transmission of information to implement the policies. As INRM indicates, conducive public atmosphere to learning (*C*_33_) is supported by effective transmission of information (*C*_34_). But SJNV’s performance in the *C*_34_ criterion is very low, and its gap value of 0.6244 is smaller only than *C*_33_. Therefore, SJNV’s improvement plans should include increasing the quality of information transmission. Coupled with strategies promoting the development of cultural and arts education, this will improve the issue at its root and help SJNV achieve its ideal standard through the mutual influences among the criteria. Presently, many creative workers at SJNV have pointed out in their interviews that poster boards are still being used by the creative community to transmit information, and paper materials such as brochures and flyers are still the main medium for transmission of information. Even though SJNV’s administrators have set up a public page on a popular social media site, the home page is not updated frequently, and the quality of publicity materials is lacking. The home page also does not support replying to comments and online enquiries. As a result, creative workers rarely use the page.

Based on the above discussions, in order to realize fundamental improvements of SJNV, this study proposes that the local government and the operators pay sufficient attention to the transmission and management of information. Currently, there is a great need to establish dedicated official website or a social media platform for SJNV, with personnel to handle the day-to-day management of the website or home page to increase the convenience and efficiency of information transmission. At the same time, multiple digital facilities should also be established in the creative community as channels to transmit information and promote the development of various cultural activities. For instance, the digital leisure and learning facilities in SJNV will most directly improve the current public environment for learning and communication. In addition, it should be noted that the internet has altered the mechanism for information production and dissemination, shifting public discussions to mobile terminals. Therefore, SJNV’s operators and community members should actively use various social media applications and explore new technologies to access the official website on mobile terminals. This will better disseminate information on the official website and enable visitors to receive timely information about activities in the creative community. Results of this study show that implementing these initiatives will have a crucial positive effect on young creative workers in SJNV in early stages of their careers. It can do so by attracting more attention and discussion on their creative work, bolstering their self-confidence.

In sum, although the performance of SJNV (performance value being 6.2162), in light of the results of this study, is “far better” than that of TCCIP (performance value being 3.8902), but seen in light of the “aspiration level gap value”, it can be discovered that SJNV still has a gap of 0.3784 that requires improvement. The performance results clearly show that SJNV, in its current condition, performs the worst in the dimension of social sustainability (*D*_3_). Further, INRM (Figure 3) also indicates that *D*_3_ is the most influential dimension in the evaluation framework of this study. This is a signal that should not be ignored. If the practical problems now discovered cannot be promptly and effectively solved, the social sustainability of the community will be severely limited and any further development will not be possible on the basis of its present condition. Therefore, the recommendation of this study is that the operational organization and management department of the community should try to solve all the major practical problems confronting SJNV under *D*_3_. In particular, the performance of SJNV in terms of the criterion of a conducive public atmosphere for learning is the least ideal. This study recommends that the creative workers in SJNV are brought in the schools or other educational organizations in the vicinity, and at the same time, in combination with the launch of cultural and artistic activities, local students and residents are invited to participate in the practices of artistic creation through providing them with spaces for display of their works and venues for interactive teaching. Further, much more crucial is that under *D*_3_, as indicated in INRM (Figure 3), high-quality transmission of information (*C*_34_) will have a critical effect on the conducive public atmosphere for learning (*C*_33_). This is exactly the starting point for devising strategies for radical improvement for SJNV. This study recommends that the information transmission channels of the creative community shall be strengthened and the speed of information update be enhanced, providing convenient, fast, and efficient community internet facilities, together with providing convenient digital leisure facilities as well as creating a digitalized learning environment at the same time.

By comparing similarities and differences between the improvement strategies of these two case communities, this study proposes that the real issues of the two creative communities should be dealt with using different standards and strategies, even though TCCIP and SJNV have many similarities (for instance, they are both located in Taichung City, whose developmental goal is to be a “creative city”; they both have a heritage of long-abandoned buildings; and they both feature urban revival strategies initiated by the local government). However, the improvement strategies and emphasis devised by this study for the two communities are completely different. In terms of the biggest issues faced by the two creative communities, TCCIP has the largest gap value in the economic sustainability (*D*_1_) dimension, while SJNV’s gap is in social sustainability (*D*_3_). Nevertheless, INRM (Figure 3) indicates that *D*_3_ is the most influential dimension in the assessment framework, while *D*_1_ is subject to the influence of the other three dimensions. Therefore, the improvement strategies devised specifically for TCCIP should emphasize improving the creative community in those criteria that have larger gap values under the *D*_2_, *D*_4_ and *D*_3_ dimensions; whereas SJNV’s emphasis of improvement should be the criteria under the *D*_3_ dimension that are close to the root influence and have larger gap values. However, the fundamental thinking and ultimate goal behind devising improvement strategies are the same: developing dynamic solution plans by systemic thinking about tackling the root influence and by realizing continuous improvement for the case studies under the limitation of available resources.

## 4. Conclusions

This study offers useful insights into the theoretical investigation and practical improvement for creative communities that can help reverse the current development crisis and achieve sustainable development. First, a systemic indicator framework has been established with reference to previous research, which can be used to assess the sustainability of creative communities. This is followed by a pre-test of the criteria, where the collected opinions of the experts are used to adjust the content of the survey questionnaire, testing the key assessment criteria. Second, the hybrid MCDM model combining DEMATEL and ANP and Modified VIKOR methods were used to evaluate the existing creative communities for proposing continuous and efficient improvement strategies for sustainability. The evaluation by this study of the sustainability of the two empirical cases is not simply an evaluation of their merits and demerits but also puts forward two profound implications: first, it breaks the myth of “relatively good.” From the results of this study, the performance of SJNV is “far better” than that of TCCIP, but from the perspective of “aspiration level gap value”, SJNV still has a gap of up to 0.3784 that requires improvement. Second, it concerns how to systematically propose an improvement strategy that “makes the good better.” From the results of this study, it can be seen that the dimension with the greatest gap value in SJNV is social sustainability (0.4784) and that with the greatest gap value in TCCIP is economic sustainability (0.6376). In the past, we might have suggested investing resources in this part of “the problem representation”. However, this study puts forward another line of thought, which is to investigate from an “influence perspective” and invest the resources in an overall package approach. For policymakers and planners, this study provides an effective way to make systematic and continuous improvement strategies for promoting creative communities toward achieving the aspiration levels in each dimension and criterion.

The limitations of this study should be acknowledged. Under the current research issue it may be more suitable to judge the assessment performances in the case studies from the perspective of the experts. But the visiting public comprises the main participants and the target groups for the various cultural activities of the creative communities. Therefore, a follow-up study could survey members of the public on the sustainability of these communities to supplement the current study. In addition, improvement strategies proposed by the research are targeted specifically at the creative communities of TCCIP and SJNV in Taichung. Therefore, these improvement policies should not be transplanted rigidly into another locality, although the process of developing improvement strategies and the evaluation frameworks provided in this study can be used as a reference for studies of other creative communities. Another issue is that the modified VIKOR method used in this study to assess performance levels in the case studies is an additive method. However, circumstances in practice may often be non-additive, and hence follow-up research may use non-additive methods to assess performance more closely approximating actual circumstances. 

## Figures and Tables

**Table 1:** Evaluation criteria and descriptions of the criteria.

Dimensions/Criteria	Descriptions	Cited References
**Economic Sustainability** (*D*_1_)		
Discovering new advantages (*C*_11_)	Completely utilize the community’s geographical advantage, consolidate current traffic resources, discover potential labor resources, and promote the coordinated development of the community and the local industries.	[24,25,26,27]
Diversification of creative talents (*C*_12_)	Attract creative talents from different fields and with different skills to come together, while emphasizing the development and training of local labor resources.	[5,14,27,28]
Increase of symbolic value (*C*_13_)	Emphasize the full utilization of tangible and intangible cultural capital, create a community image that is healthy, vibrant and attractive, and encourages marketing activities that promote community resources and cultural characteristics.	[6,12,29,30]
Continuous innovation of products (*C*_14_)	Increase the innovative output of creative communities and enhance the differentiation and quality of creative products and services.	[31,32,33,34]
Stable financial base (*C*_15_)	Establish tax programs for cities to raise funds for culture and the arts, and promote funding by local governments and relevant enterprises to develop cultural and arts industries, and help implement community activities.	[22,23,31,32,36]
**Environmental Sustainability** (*D*_2_)		
Energy conservation and emission reduction in buildings (*C*_21_)	Maximize the efficient use of energy, minimize the demand for fossil fuels, adopt planning that responds to microclimates, fully utilize natural ventilation and lighting, and emphasize the development and utilization of renewable energy.	[37,38,39,40]
Recycling energy (*C*_22_)	Emphasize the reassessment of waste, advocate designs that use upcycling, and transform waste material into new materials or products or those of higher environmental value.	[41,42]
Biodiversity (*C*_23_)	Defend and rehabilitate habitats and wetlands, protect wild animals, construct eco-corridors, and enhance the connective and ecological functions of the green system, providing vegetation and facilities that attract birds and butterflies.	[5,9,35]
High-quality constructed environment (*C*_24_)	Make the physical environment of the community greener on multiple levels and cultivate native plants, while encouraging the renewal of public open spaces and infrastructures and creating new urban spaces and environmental symbols.	[5,9,27,43]
Unique spatial environment (*C*_25_)	Utilize community resources to create local characteristics, enhance the synergy between the creation of creative communities and local physical landscapes, and create a spatial environment and landscape imagery that features vivid characteristics that are appreciated by creative workers and other members of the community.	[24,27,29,42]
**Social Sustainability** (*D*_3_)		
Highly effective civic participation mechanism (*C*_31_)	Encourage community members to express their opinions, provide sufficient opportunities for civic participation, allow the community to participate willingly, and express their views regarding various decisions and public matters in their local creative communities and contribute their valuable design proposals.	[4,5,8,21,29,44]
Widespread interaction and communication (*C*_32_)	Provide highly accessible and convenient community public spaces and community service facilities, enhance the safety and comfort of public spaces and activity premises, and promote the widespread interaction and communication between different levels of society.	[8,9,12,45,46]
Conducive public atmosphere to learning (*C*_33_)	Fully utilize public spaces in the community to create more opportunities for learning and interaction and to allow creative workers to showcase their crafts and disseminate their knowledge in a more direct and effective manner.	[9,12,47]
High-quality transmission of information (*C*_34_)	Provide convenient and efficient internet facilities in the community, enrich the channels for dissemination of information, increase the speed of information update, and enhance the accuracy of disseminated information in the community.	[8,32]
Sound welfare system (*C*_35_)	Provide adequate care and support to low-income creative workers and other disadvantaged groups in the creative communities, safeguard their quality of living and working rights, and reject all forms of discrimination.	[9,21,35]
**Cultural Sustainability** (*D*_4_)		
Amalgamation of diverse cultures (*C*_41_)	Respect and preserve the different cultural backgrounds, ways of thinking and belief systems of different ethnic communities, and promote the communication and concerted development of multiple cultures.	[5,15,48]
Self-initiation of cultural activities (*C*_42_)	Promote the widespread implementation of cultural and arts events in creative communities, emphasize a bottom-up approach in implementing community activities, encourage self-initiated expressions in civic societies, and stimulate passionate participation of members of creative communities.	[5,8,9,35]
Preservation of cultural heritage (*C*_43_)	Emphasize the preservation and reuse of cultural heritage properties such as historical buildings or cultural and geographical features and enhance non-material cultural heritage.	[5,9,13,15,48]

**Table 2:** The performance evaluation of the case study using VIKOR.

Dimensions/Criteria	Influential Weights (IWs)		Taichung Cultural and Creative Industry Park (TCCIP)		Shen Ji New Village (SJNV)	
	Local Weights	Global Weights	Performance $f_{1j}$	Gap Ratio $r_{1j}\;and\;s_{1}$	Performance $f_{2j}$	Gap Ratio $r_{2j}\;and\;s_{2}$
**Economic Sustainability (*D*_1_)**	**0.3170**		**3.6243**	**0.6376**	**6.7653**	**0.3235**
Discovering new advantages (*C*_11_)	0.1669	0.0529	5.1944	0.4806	5.0000	0.5000
Diversification of creative talents (*C*_12_)	0.2208	0.0700	1.6111	0.8389	7.9556	0.2044
Increase of symbolic value (*C*_13_)	0.2363	0.0749	4.0000	0.6000	6.4889	0.3511
Continuous innovation of products(*C*_14_)	0.2019	0.0640	4.0278	0.5972	6.0667	0.3933
Stable financial base (*C*_15_)	0.1741	0.0552	3.6944	0.6306	8.1333	0.1867
Environmental Sustainability (*D*_2_)	**0.2240**		**3.8243**	**0.6176**	**5.6379**	**0.4362**
Energy conservation and emission reduction in buildings (*C*_21_)	0.1429	0.0320	4.4167	0.5583	5.2222	0.4778
Recycling energy (*C*_22_)	0.1419	0.0318	3.8056	0.6194	5.3556	0.4644
Biodiversity (*C*_23_)	0.1835	0.0411	4.0278	0.5972	5.0667	0.4933
High-quality constructed environment (*C*_24_)	0.2540	0.0569	5.1667	0.4833	4.2000	0.5800
Unique spatial environment (*C*_25_)	0.2777	0.0622	2.1667	0.7833	7.6889	0.2311
Social Sustainability (*D*_3_)	**0.1951**		**4.1553**	**0.5845**	**5.2158**	**0.4784**
Highly effective civic participation mechanism (*C*_31_)	0.1466	0.0286	3.5833	0.6417	6.3778	0.3622
Widespread interaction and communication (*C*_32_)	0.2030	0.0396	2.8889	0.7111	5.5778	0.4422
Conducive public atmosphere to learning (*C*_33_)	0.2711	0.0529	3.8889	0.6111	2.8889	0.7111
High-quality transmission of information (*C*_34_)	0.1492	0.0291	5.7500	0.4250	3.7556	0.6244
Sound welfare system (*C*_35_)	0.2301	0.0449	4.9167	0.5083	7.8444	0.2156
Cultural Sustainability (*D*_4_)	**0.2639**		**4.0697**	**0.5930**	**6.7872**	**0.3213**
Amalgamation of diverse cultures (*C*_41_)	0.3831	0.1011	3.3889	0.6611	5.9778	0.4022
Self-initiation of cultural activities (*C*_42_)	0.3005	0.0793	2.5833	0.7417	8.1333	0.1867
Preservation of cultural heritage (*C*_43_)	0.3164	0.0835	6.3056	0.3694	6.4889	0.3511
Total Performance			**3.8902**		**6.2162**	
Total Gap				**0.6110**		**0.3784**

Note: Gap ratio $r_{kj}=(|f_{j}^{aspired}-f_{kj}|)/(|f_{j}^{aspired}-f_{j}^{worst}|)=(10-f_{kj})/(10-0),$ $s_{k}=\sum_{j=1}^{n}w_{j}r_{kj},$ where $f_{kj}$ denotes j criterion of k alternative, i.e., k=1 (TCCIP) and k=2 (SJNV).

## Appendix A. Research Steps

The D-DANP-mV model, which was adopted for this study, was proposed by Tzeng [62], who combined new concepts and techniques to solve complex and dynamic real-world problems. This hybrid modified MADM model included three analysing tools: the DEMATEL technique, the DANP technique, and the modified VIKOR method.

### Appendix A.1. DEMATEL Technique

*Step 1: Establish the direct influence relation matrix **E**.* Data were obtained using the questionnaire, and the scales used in the questionnaire involved an integer score of 0, 1, 2, 3, or 4, ranging from 0 representing absolutely no influence to 4 representing very high influence, according to natural language criteria from linguistics. The respondent had to be an expert in the field and use the pairwise comparison method to evaluate the degree of influence of the criteria, and to show the degree to which each criterion *i* affects each criterion *j*. This matrix must be a n×n nonnegative matrix. According to the results from *H* experts, the direct influence relation matrix ***E*** is shown in Equation (A1), and the direct influence relation matrix from all experts is $E^{h}={\left[e_{ij}^{h}\right]}_{n\times n},$ h=1,2,…,H, where $E^{1},\ldots ,E^{h},\ldots ,E^{H}$:

(A1) $$E=\left[\begin{array}{ccccc} e_{11} & \cdots & e_{1j} & \cdots & e_{1n}\\ \vdots & & \vdots & & \vdots \\ e_{i1} & \cdots & e_{ij} & \cdots & e_{in}\\ \vdots & & \vdots & & \vdots \\ e_{n1} & \cdots & e_{nj} & \cdots & e_{nn} \end{array}\right]$$

*Step 2: Constitute the average direct influence relation matrix **A**.* The average scores of the *H* experts are $\begin{array}{l} a_{ij}=\frac{1}{H}\sum_{h=1}^{H}e_{ij}^{h} \end{array}$. The average matrix is called the average direct influence relation matrix ***D*** and represents the degree of influence that one criterion exerts on another and the degree of influence that the criterion receives from another, as shown in Equation (A2):

(A2) $$A=\left[\begin{array}{ccccc} a_{11} & \cdots & a_{1j} & \cdots & a_{1n}\\ \vdots & & \vdots & & \vdots \\ a_{i1} & \cdots & a_{ij} & \cdots & a_{in}\\ \vdots & & \vdots & & \vdots \\ a_{n1} & \cdots & a_{nj} & \cdots & a_{nn} \end{array}\right].$$

*Step 3: Examine consensus.* The value of consensus can be estimated by Equation (A3), which represents the level of experts’ consensus. The threshold of the average gap ratio is 5% in statistics; a value less than 5% implies a confidence level above 95%, which also represents a stable system. Conversely, if an unstable system is obtained, the first phase should be implemented again to verify whether data collection is correct and whether the number of experts is sufficient:

(A3) $$\mathrm{Average}\;\mathrm{gap}-\mathrm{ratio}\;\mathrm{in}\;\mathrm{consensus}(\%)=\frac{1}{n(n-1)}\sum_{i=1}^{n}\sum_{j=1}^{n}\left(\left|a_{ij}^{H}-a_{ij}^{H-1}\right|/a_{ij}^{H}\right)\times 100\%$$

*Step 4: Formulate the normalized average direct influence relation matrix **D**.* The normalized average direct influence relation matrix ***D*** is acquired by normalizing the matrix ***A***. The matrix ***D*** is easily derived from Equations (A4) and (A5), in which all principal diagonal criteria are equal to 0:

(A4) D=b·A

(A5) $$b=\min\left\{\frac{1}{\max_{1\;\leq i\leq n}\sum_{j=1}^{n}a_{ij}},\frac{1}{\max_{1\;\leq j\leq n}\sum_{i=1}^{n}a_{ij}}\right\}$$

*Step 5: Construct the total influence relation matrix* ***T****.* A continuous decrease of the indirect effects of problems moves with the powers of the matrix D, e.g., $D^{2},\ldots ,D^{\infty },$ and $\lim_{q\to \infty }D^{q}={\left[0\right]}_{n\times n},$ for $\lim_{q\to \infty }(I+D+D^{2}+\ldots +D^{q})=\;{\left(I-D\right)}^{-1}$, where I is a n×n unit matrix. The total influence relation matrix ***T*** is a n×n matrix and is defined by $T={\left[t_{ij}\right]}_{n\times n},\;i,j=1,\;2,\;\ldots ,\;n$, as shown in Equation (A6):

(A6) $$T=D+D^{2}+\ldots +D^{q}=D(I+D\;+\;D^{2}+\ldots +D^{q-1})=D(I+\;D\;+\;D^{2}+\ldots +D^{q-1})(I-D){\left(I-D\right)}^{-1}\;=D{\left(I-D\right)}^{-1},\mathrm{when}\lim_{q\to \infty }D^{q}={\left[0\right]}_{n\times n}$$

*Step 6: Illustration of INRM.* The total influence relation matrix ***T*** of INRM can be acquired using Equations (A7) and (A8) to generate each row sum and column sum in the matrix ***T***, respectively:

(A7) $$o={\left(o_{i}\right)}_{n\times 1}={\left[\sum_{j=1}^{n}t_{ij}\right]}_{n\times 1}=(o_{1},\ldots ,o_{i},\ldots ,o_{n})$$

(A8) $$r={\left(r_{i}\right)}_{n\times 1}={(r_{j}{)}^{\prime }}_{1\times n}={\left[\sum_{i=1}^{n}t_{ij}\right]}^{\prime }{}_{1\times n}=(r_{1},\ldots ,r_{j},\ldots ,r_{n}{)}^{\prime },$$

where $o_{i}$ is the sum of a row in the total influence relation matrix ***T***, which represents the total effects (both direct and indirect) of criterion/perspective *i* on all other criteria/perspectives ${\left[\sum_{j=1}^{n}t_{ij}\right]}_{n\times 1}$. Similarly, $r_{j}$ is the column sum in the total influence relation matrix ***T***, which represents the total effects (both direct and indirect) of criterion/perspective *j* received from all other criteria/perspectives ${\left[\sum_{i=1}^{n}t_{ij}\right]}^{\prime }{}_{1\times n}$. $\left(o_{i}+r_{i}\right)$ provides an index of the strength of the total influences given and received; that is, $\left(o_{i}+r_{i}\right)$ indicates the degree of importance of the criterion/perspective *i* in the system. In addition, $\left(o_{i}-r_{i}\right)$ provides an index of the degree of the cause of the total influence. If $\left(o_{i}-r_{i}\right)$ is positive, then criterion/perspective *i* is a net causer, and if $\left(o_{i}-r_{i}\right)$ is negative, then criterion/perspective *i* is a net receiver.

*Step 7: Construct the total influence relation matrix of criteria* $T_{C}$ *and dimension* $T_{D}$*.* The total influence relation matrix of criteria $T_{C}$ from each perspective (dimension or cluster), with different degrees of influence relation for the criteria, is shown in Equation (A9), where $\sum_{j=1}^{m}m_{j}=n$, m<n, and $T_{c}^{ij}$ as a $m_{i}\times m_{j}$ matrix:

(A9) $$T_{C}=\begin{array}{c} \begin{array}{l} D_{1}\\ \vdots \end{array}\\ \begin{array}{l} D_{i}\\ \vdots \end{array}\\ D_{m} \end{array}\begin{array}{c} c_{11}\\ c_{12}\\ \vdots \\ c_{1m_{1}}\\ \vdots \\ c_{i1}\\ c_{i2}\\ \vdots \\ c_{im_{i}}\\ \vdots \\ c_{m1}\\ c_{m2}\\ \vdots \\ c_{mm_{m}} \end{array}{\overset{\begin{array}{ccc} \begin{array}{ccc} \begin{array}{ccc} \begin{array}{ccc} \;D_{1}\; & & \;D_{j} \end{array} & & \end{array} & & \end{array} & D_{m} & \end{array}}{\overset{\begin{array}{ccccc} \;c_{11\cdots }c_{1m_{1}} & \cdots & c_{j1\cdots }c_{jm_{{}_{j}}} & \cdots & c_{m1\cdots }c_{mm_{m}} \end{array}}{\left[\begin{array}{ccccc} T_{c}^{11} & \cdots & T_{c}^{1j} & \cdots & T_{c}^{1m}\\ \vdots & & \vdots & & \vdots \\ T_{c}^{i1} & \cdots & T_{c}^{ij} & \cdots & T_{c}^{im}\\ \vdots & & \vdots & & \vdots \\ T_{c}^{m1} & \cdots & T_{c}^{mj} & \cdots & T_{c}^{mm} \end{array}\right]}}}_{n\times n|m<n,\;\sum_{j=1}^{m}m_{j}=n},$$

where $D_{m}$ is the *m*th cluster; $c_{mm}$ is the *m*th criterion in the *m*th dimension; and $T_{c}^{ij}$ is a submatrix of the influence relation for the criteria from a comparison of the *i*th dimension with the *j*th dimension. In addition, if the *i*th dimension has no influence on the *j*th dimension, then submatrix $T_{c}^{ij}=[0]$, demonstrating the independence (no influence relation) of each criterion on other criteria. Moreover, the total influence relation matrix of dimension $T_{D}$ is shown in Equation (A10):

(A10) $$T_{D}^{}={\left[\begin{array}{ccccc} t_{11}^{} & \cdots & t_{1j}^{} & \cdots & t_{1m}^{}\\ \vdots & & \vdots & & \vdots \\ t_{i1}^{} & \cdots & t_{ij}^{} & \cdots & t_{im}^{}\\ \vdots & & \vdots & & \vdots \\ t_{m1}^{} & \cdots & t_{mj}^{} & \cdots & t_{mm}^{} \end{array}\right]}_{m\times m}.$$

The D-DANP-mV model is required to identify the dimensions and criteria individually while proposing an improvement strategy; thus, drawing the INRM of dimensions and criteria should be divided into the matrix $T_{C}$ and the matrix $T_{D}$.

### Appendix A.2. DANP Technique

*Step 1: Calculate the unweighted supermatrix* $W_{}^{\alpha }$. Normalize the total influence relation matrix $T_{C}$ by dimensions (called “clusters”) as shown in Equation (A11):

(A11) $$T_{C}^{\alpha }=\begin{array}{c} \begin{array}{l} D_{1}\\ \vdots \end{array}\\ \begin{array}{l} D_{i}\\ \vdots \end{array}\\ D_{m} \end{array}\begin{array}{c} c_{11}\\ c_{12}\\ \vdots \\ c_{1m_{1}}\\ \vdots \\ ci1\\ c_{i2}\\ \vdots \\ c_{im_{i}}\\ \vdots \\ c_{m1}\\ c_{m2}\\ \vdots \\ c_{mm_{m}} \end{array}{\overset{\begin{array}{ccc} \begin{array}{ccc} \begin{array}{ccc} \begin{array}{ccc} \;D_{1}\; & & \text{​ }D_{j}\; \end{array} & & \end{array} & & \end{array} & D_{m} & \end{array}\;}{\overset{\begin{array}{ccccc} \;c_{11\cdots }c_{1m_{1}} & \ldots & c_{j1\cdots }c_{jm_{j}}\; & \cdots & c_{n1\cdots }c_{mm_{m}}\text{ ​ } \end{array}}{\left[\begin{array}{ccccc} T_{c}^{\alpha 11} & \cdots & T_{c}^{\alpha 1j} & \cdots & T_{c}^{\alpha 1m}\\ \vdots & & \vdots & & \vdots \\ T_{c}^{\alpha i1} & \cdots & T_{c}^{\alpha ij} & \cdots & T_{c}^{\alpha im}\\ \vdots & & \vdots & & \vdots \\ T_{c}^{\alpha m1} & \cdots & T_{c}^{\alpha mj} & \cdots & T_{c}^{\alpha mm} \end{array}\right]}}}_{n\times n|m<n,\;\sum_{j=1}^{m}m_{j}=n},$$

where $T_{C}^{\alpha }$ denotes the normalizing total influence relation matrix of criteria by dimensions, and $T_{c}^{\alpha 14}$ is derived from Equations (A12) and (A13). Similarly, $T_{c}^{\alpha mm}$ can be obtained:

(A12) $$t_{i}^{14}=\sum_{j=1}^{m_{4}}t_{ij}^{14},i=1,2,\cdots ,m_{1}$$

(A13) $$T_{c}^{\alpha 14}=\begin{array}{c} \;c_{11}\;\\ \;\vdots \;\\ \;c_{1i}\;\\ \;\vdots \;\\ \;c_{1m_{1}}\; \end{array}\begin{array}{c} \begin{array}{ccccccccc} c_{4}{}_{1}\; & & \cdots & & c_{4}{}_{j} & & \;\cdots \; & & \;c_{4m_{4}} \end{array}\\ \left[\begin{array}{ccccc} t_{11}^{14}/t_{1}^{14} & \cdots & t_{1j}^{14}/t_{1}^{14} & \cdots & t_{1m_{4}}^{14}/t_{1}^{14}\\ \vdots & & \vdots & & \vdots \\ t_{i1}^{14}/t_{i}^{14} & \cdots & t_{ij}^{14}/t_{i}^{14} & \cdots & t_{im_{4}}^{14}/t_{i}^{14}\\ \vdots & & \vdots & & \vdots \\ t_{m_{1}1}^{14}/t_{m_{1}}^{14} & \cdots & t_{m_{1}j}^{14}/t_{m_{1}}^{14} & \cdots & t_{m_{1}m_{4}}^{14}/t_{m_{1}}^{14} \end{array}\right] \end{array}=\begin{array}{c} \left[\begin{array}{ccccc} t_{11}^{\alpha 14} & \cdots & t_{1j}^{\alpha 14} & \cdots & t_{1m_{4}}^{\alpha 14}\\ \vdots & & \vdots & & \vdots \\ t_{i1}^{\alpha 14} & \cdots & t_{ij}^{\alpha 14} & \cdots & t_{im_{4}}^{\alpha 14}\\ \vdots & & \vdots & & \vdots \\ t_{m_{1}1}^{\alpha 14} & \cdots & t_{m_{1}j}^{\alpha 14} & \cdots & t_{m_{1}m_{4}}^{\alpha 14} \end{array}\right] \end{array}.$$

According to the pairwise comparisons of the criteria and on the basis of the basic concept of ANP, the unweighted supermatrix $W_{}^{\alpha }$ can be obtained by transposing the normalized influence relation matrix $T_{C}^{\alpha }$ by dimensions (clusters), that is, $W^{\alpha }=(T_{C}^{\alpha }{)}^{\prime }$, as shown in Equation (A14):

(A14) $$W^{\alpha }=(T_{C}^{\alpha }{)}^{\prime }=\begin{array}{c} \begin{array}{l} D_{1}\\ \vdots \end{array}\\ \begin{array}{l} D_{j}\\ \vdots \end{array}\\ D_{m} \end{array}\begin{array}{c} \begin{array}{l} c_{11}\\ c_{\begin{array}{l} 12\\ \vdots \end{array}}\\ c_{\begin{array}{l} 1m_{1}\\ \vdots \end{array}}\\ c_{j1}\\ c_{\begin{array}{l} j2\\ \vdots \end{array}} \end{array}\\ \begin{array}{l} c_{\begin{array}{l} jm_{j}\\ \vdots \end{array}}\\ c_{m1}\\ c_{\begin{array}{l} m2\\ \vdots \end{array}}\\ c_{mm_{m}} \end{array} \end{array}{\overset{\begin{array}{ccc} \begin{array}{ccc} \begin{array}{ccc} \begin{array}{ccc} \;D_{1}\; & & \text{​ }D_{i}\; \end{array} & & \end{array} & & \end{array} & D_{m} & \end{array}\;}{\overset{\begin{array}{ccccc} \;c_{11\cdots }c_{1m_{1}}\; & \;\ldots & c_{i1\cdots }c_{im_{i}}\; & \;\cdots & c_{m1\cdots }c_{mm_{m}}\; \end{array}}{\left[\begin{array}{ccccc} W^{11} & \cdots & W^{i1} & \cdots & W^{m1}\\ \vdots & & \vdots & & \vdots \\ W^{1j} & \cdots & W^{ij} & \cdots & W^{mj}\\ \vdots & & \vdots & & \vdots \\ W^{1m,} & \cdots & W^{im} & \cdots & W^{mm} \end{array}\right]}}}_{n\times n|m<n,\;\sum_{j=1}^{m}m_{j}=n}.$$

*Step 2: Calculate weighted supermatrix*. The normalized total influence-relation matrix of dimensions $T_{D}^{\alpha }$ can be obtained through the total influence-relation matrix $T_{D}^{}$ divided by $d_{i}=\sum_{j=1}^{m}t_{ij}$, i=1,2,…,m, as shown in Equation (A15):

(A15) $${Τ}_{D}^{\alpha }={\left[\begin{array}{ccccc} t_{11}/d_{1} & \cdots & t_{1j}/d_{1} & \cdots & t_{1m}/d_{1}\\ \vdots & & \vdots & & \vdots \\ t_{i1}/d_{i} & \cdots & t_{ij}/d_{i} & \cdots & t_{im}/d_{i}\\ \vdots & & \vdots & & \vdots \\ t_{m1}/d_{m} & \cdots & t_{mj}/d_{m} & \cdots & t_{mm}/d_{m} \end{array}\right]}_{m\times m}={\left[\begin{array}{ccccc} t_{11}^{\alpha D} & \cdots & t_{1j}^{\alpha D} & \cdots & t_{1m}^{\alpha D}\\ \vdots & & \vdots & & \vdots \\ t_{i1}^{\alpha D} & \cdots & t_{ij}^{\alpha D} & \cdots & t_{im}^{\alpha D}\\ \vdots & & \vdots & & \vdots \\ t_{m1}^{\alpha D} & \cdots & t_{mj}^{\alpha D} & \cdots & t_{mm}^{\alpha D} \end{array}\right]}_{m\times m}.$$

The matrix $T_{D}^{\alpha }$ and the unweighted supermatrix $W_{}^{\alpha }$ and the weighted supermatrix ***W*** can be easily obtained by Equation (A16), where $t_{ij}^{\alpha D}$ is a scalar and $\sum_{j=1}^{m}m_{j}=n$:

(A16) $$W=T_{D}^{\alpha }W^{\alpha }=\begin{array}{c} \begin{array}{l} D_{1}\\ \vdots \end{array}\\ \begin{array}{l} D_{j}\\ \vdots \end{array}\\ D_{m} \end{array}\begin{array}{c} \begin{array}{l} c_{11}\\ c_{\begin{array}{l} 12\\ \vdots \end{array}}\\ c_{\begin{array}{l} 1m_{1}\\ \vdots \end{array}}\\ c_{j1}\\ c_{\begin{array}{l} j2\\ \vdots \end{array}} \end{array}\\ \begin{array}{l} c_{\begin{array}{l} jm_{j}\\ \vdots \end{array}}\\ c_{m1}\\ c_{\begin{array}{l} m2\\ \vdots \end{array}}\\ c_{mm_{m}} \end{array} \end{array}\overset{\begin{array}{ccc} \begin{array}{ccc} \begin{array}{ccc} \begin{array}{ccc} \;D_{1}\; & \; & \text{​ }D_{i}\; \end{array} & \; & \end{array} & & \end{array}\; & D_{m} & \end{array}\;}{\overset{\begin{array}{ccccc} \;c_{11\cdots }c_{1m_{1}} & \ldots \; & c_{i1\cdots }c_{im_{i}}\; & \;\cdots & c_{m1\cdots }c_{mm_{m}}\; \end{array}}{\left[\begin{array}{ccccc} t_{11}^{\alpha D}\times W^{11} & \cdots & t_{i1}^{\alpha D}\times W^{i1} & \cdots & t_{m1}^{\alpha D}\times W^{m1}\\ \vdots & & \vdots & & \vdots \\ t_{1j}^{\alpha D}\times W^{1j} & \cdots & t_{ij}^{\alpha D}\times W^{ij} & \cdots & t_{mj}^{\alpha D}\times W^{mj}\\ \vdots & & \vdots & & \vdots \\ t_{1m}^{\alpha D}\times W^{1m} & \cdots & t_{im}^{\alpha D}\times W^{im} & \cdots & t_{mm}^{\alpha D}\times W^{mm} \end{array}\right]}}.$$

*Step 3: Limit weighted supermatrix*. Limit the weighted supermatrix by raising it to the *z*th power until the supermatrix has converged and become a stable supermatrix. The global priority vectors, global weight $w^{g}$—which are called the relative IWs of DANP—are obtained, such as $lim_{z\to \infty }{\left(W\right)}^{z}$, where *z* represents any number of power. By summing the relative IWs of each criterion in every dimension, the local weights of dimension $w_{D}^{l}$ can be obtained. Subsequently, the global weights of each criterion can be divided by the local weights of its own dimension to yield the local weights of criteria $w_{c}^{l}$.

### Appendix A.3. Modified VIKOR Method

*Step 1: Derive the positive ideal solution and the negative ideal solution replaced by the aspiration levels and the worst value to fit the current real-world situation*. Define the best value (aspiration level) shown as $f_{j}^{aspired}$ in *j* criterion and the worst value $f_{j}^{worst}$ for all criteria, which can be acquired from the traditional form to the modified form.

(1) The traditional approach for deriving the positive ideal solution and negative ideal solution is as follows:
  The positive ideal solution: $f^{*}=(f_{1}^{*},\ldots ,f_{j}^{*},\ldots ,f_{n}^{*}),$ where $f_{j}^{*}=\max_{k}\;\{f_{kj}|k=1,2,\ldots ,b\}$;
  The negative ideal solution: $f^{-}=(f_{1}^{-},\ldots ,f_{j}^{-},\ldots ,f_{n}^{-}),$ where $f_{j}^{-}=\min_{k}\;\{f_{kj}|k=1,2,\ldots ,b\}.$ This approach can only be used for the ranking and selection of alternatives and must be used for more than two alternatives, $f_{j}^{*}=\max_{k}\;f_{kj}$ and $f_{j}^{-}=\min_{k}\;f_{kj}$ when k=1,2,…,b.
(2) The modified approach for replacement by the aspiration level and the worst value is, as follows:
  The aspiration level: $f_{}^{aspired}=(f_{1}^{aspired},\ldots ,f_{j}^{aspired},\ldots ,f_{n}^{aspired})$, where $f_{j}^{aspired}$ is an aspiration level, or called the best value;
  The worst values: $f_{}^{worst}=(f_{1}^{worst},\ldots ,f_{j}^{worst},\ldots ,f_{n}^{worst})$, where $f_{j}^{worst}$ is a worst value.

In this study, performance scores ranging from 0 to 10 (very bad ← 0, 1, 2, …, 9, 10 → very good) are used with natural language in the linguistic/semantic questionnaire; thus, the aspiration level takes the highest score of 10 and the worst value takes the value of 0. Hence, $f_{j}^{aspired}=10$ is defined as the aspiration level and $f_{j}^{worst}=0$ as the worst value.

*Step 2: Determine the mean group utility for the gap and then establish the priority improvement strategy*. These values can be calculated using Equation (A17):

(A17) $$s_{k}=\sum_{j=1}^{n}w_{j}r_{kj}=\sum_{j=1}^{n}w_{j}(|f_{j}^{aspired}-f_{kj}|)/(|f_{j}^{aspired}-f_{j}^{worst}|),$$

where $s_{k}$ is defined as the normalized ratio (%) of distance to the aspiration level, which implies the synthesized gap of the criteria. Here, $w_{j}$ indicates the IWs for the criteria obtained from DANP.

## Appendix B. Results in Detail

DANP (DEMATEL-based ANP) was used to develop the structure of influential relationships and analyze the relationship (interaction) and the influential degree of the four dimensions and 18 criteria. As mentioned in Appendix A, we used the results of the fifteen experts’ questionnaires to measure the total influence matrix ***T*** and the degrees of influence. Table A1 shows the initial influence matrix $A={\left[a_{ij}\right]}_{n\times n}$ derived from the average value of the fifteen experts’ questionnaires. The consistency gaps of these fifteen questionnaires were 3.16% and were smaller than 5%, implying that the confidence level was 96.84%, which is more than 95%, as demonstrated by the following text.

Table A2, Table A3 and Table A4 show the relationship between the total influence matrix ***T*** and the degrees of influence. Table A2 illustrates the total relationships among the 18 criteria. Table A3 illustrates the two types of information, namely the relationships among the three dimensions and the degree of influence of each dimension. The degree of influence of each dimension combined with the INRM revealed that, based on experts’ judgment, the dimension of social sustainability (*D*_3_) had the highest effect among all dimensions and was thus the most influential dimension. The degree of influence of economic sustainability (*D*_1_) was the lowest, cultural sustainability (*D*_4_) and environmental sustainability (*D*_2_) had intermediate influence. Table A4 presents the sum of influences given or received from the degree of influence of each criterion within its own dimension.

The IWs of criteria were obtained from the ANP process in the DANP model. First, based on Equations (A9)–(A12), the unweighted supermatrix $W_{}^{\alpha }$ was obtained by transposing the normalized influence matrix $T_{C}$, as shown in Table A5. The weighted supermatrix W based on Equations (A13) and (A14) is shown in Table A6. Then, the relative IWs of DANP were obtained by limiting the power of the weighted supermatrix, as shown in Table A7. The criterion of amalgamation of diverse cultures (*C*_41_) exhibited the highest IW value (0.1011), whereas highly effective civic participation mechanism (*C*_31_) exhibited the lowest value (0.0286).

**Table A1:** Initial influence matrix *A*.

Criteria	*C*_11_	*C*_12_	*C*_13_	*C*_14_	*C*_15_	*C*_21_	*C*_22_	*C*_23_	*C*_24_	*C*_25_	*C*_31_	*C*_32_	*C*_33_	*C*_34_	*C*_35_	*C*_41_	*C*_42_	*C*_43_
*C*_11_	0.000	3.133	2.867	3.267	2.933	1.867	2.800	2.667	2.800	3.000	0.933	2.000	2.267	1.267	1.533	3.467	0.667	2.400
*C*_12_	2.067	0.000	2.133	3.400	1.867	0.467	0.733	0.600	1.333	1.200	1.267	1.800	2.600	0.733	2.133	2.667	1.733	1.133
*C*_13_	1.733	2.800	0.000	1.600	3.067	0.200	0.333	0.600	1.733	1.600	0.400	0.600	1.067	0.733	0.133	2.600	2.200	0.933
*C*_14_	1.467	1.133	3.067	0.000	2.933	0.533	1.067	1.133	1.800	2.467	0.533	0.733	1.733	1.133	0.333	1.467	1.400	3.000
*C*_15_	1.467	3.067	2.133	1.733	0.000	1.333	1.000	1.733	2.800	1.467	1.400	1.867	2.000	1.067	3.067	2.333	3.200	2.667
*C*_21_	0.467	0.267	2.333	1.267	1.533	0.000	2.467	2.733	2.333	2.667	0.133	0.133	0.333	0.267	0.933	0.067	0.067	0.600
*C*_22_	2.733	0.733	2.200	2.333	2.000	3.800	0.000	2.467	2.267	2.600	0.133	0.067	0.400	0.200	1.267	0.133	0.267	0.200
*C*_23_	1.267	1.733	3.000	1.467	0.333	1.533	1.333	0.000	3.333	2.800	0.400	0.267	0.667	0.200	0.800	0.733	0.200	0.867
*C*_24_	1.667	3.200	2.867	1.733	1.400	0.933	0.933	2.400	0.000	2.333	0.467	3.067	3.467	1.333	2.533	0.467	2.200	0.933
*C*_25_	1.467	3.200	3.333	1.467	2.800	0.733	1.467	1.333	3.400	0.000	0.267	0.667	1.400	0.333	0.267	1.933	0.400	1.400
*C*_31_	3.133	2.667	2.067	2.800	3.067	2.000	1.800	2.267	2.667	3.000	0.000	3.200	2.467	1.000	3.400	1.333	3.333	3.467
*C*_32_	3.200	0.467	2.133	3.400	2.600	0.933	0.667	0.667	1.000	1.667	2.067	0.000	2.733	2.533	1.267	3.067	2.200	2.600
*C*_33_	3.600	3.600	2.933	3.400	3.333	1.267	1.600	2.000	1.733	3.000	3.733	3.733	0.000	2.600	3.067	3.667	3.667	3.400
*C*_34_	2.467	3.200	2.800	2.467	3.133	1.733	1.733	1.533	0.467	1.800	2.733	3.400	3.533	0.000	2.533	3.067	3.533	3.267
*C*_35_	0.400	3.000	1.933	1.733	0.600	0.200	0.333	1.867	1.400	1.000	1.733	0.467	1.267	0.800	0.000	3.467	1.467	0.467
*C*_41_	1.133	3.867	3.667	3.467	0.600	0.600	0.933	1.800	1.533	3.067	0.333	0.600	2.200	0.867	2.400	0.000	2.067	2.067
*C*_42_	2.400	3.267	3.533	3.467	1.733	1.200	0.533	0.667	1.067	1.467	1.933	3.667	2.400	2.600	2.800	3.667	0.000	3.400
*C*_43_	3.600	3.067	3.400	1.600	1.267	2.600	0.867	0.200	2.733	3.800	0.467	0.267	1.667	0.200	0.933	3.533	1.867	0.000

Note: $\mathrm{Average}\;\mathrm{gap}-\mathrm{ratio}\;\mathrm{in}\;\mathrm{consensus}\;(\%)=\frac{1}{n(n-1)}\sum_{i=1}^{n}\sum_{j=1}^{n}\left(\left|a_{ij}^{H}-a_{ij}^{H-1}\right|/a_{ij}^{H}\right)\times 100\%=3.16\%<5\%$, where *n* is the number of criteria (*n* = 18), *H* is the sample of fifteen experts (*H* = 15) whose practical experience and significant confidence reach 96.84% (more than 95%).

**Table A2:** The total influence matrix of criteria *T*_C_.

Criteria	*C*_11_	*C*_12_	*C*_13_	*C*_14_	*C*_15_	*C*_21_	*C*_22_	*C*_23_	*C*_24_	*C*_25_	*C*_31_	*C*_32_	*C*_33_	*C*_34_	*C*_35_	*C*_41_	*C*_42_	*C*_43_
*C*_11_	0.075	0.156	0.159	0.152	0.134	0.082	0.098	0.109	0.132	0.144	0.058	0.094	0.115	0.064	0.093	0.150	0.081	0.117
*C*_12_	0.096	0.074	0.119	0.134	0.096	0.042	0.047	0.055	0.083	0.087	0.057	0.079	0.105	0.047	0.090	0.118	0.087	0.079
*C*_13_	0.079	0.115	0.064	0.087	0.106	0.031	0.033	0.046	0.080	0.082	0.034	0.049	0.067	0.040	0.044	0.104	0.085	0.064
*C*_14_	0.081	0.091	0.130	0.061	0.111	0.042	0.051	0.060	0.089	0.106	0.039	0.055	0.083	0.050	0.051	0.089	0.075	0.107
*C*_15_	0.096	0.147	0.134	0.116	0.069	0.066	0.058	0.084	0.121	0.105	0.065	0.090	0.105	0.059	0.117	0.124	0.123	0.116
*C*_21_	0.041	0.047	0.090	0.061	0.064	0.021	0.067	0.079	0.082	0.089	0.018	0.025	0.036	0.021	0.043	0.035	0.029	0.040
*C*_22_	0.090	0.067	0.101	0.092	0.083	0.098	0.028	0.083	0.091	0.099	0.023	0.031	0.046	0.025	0.057	0.047	0.040	0.042
*C*_23_	0.062	0.083	0.111	0.073	0.048	0.052	0.049	0.030	0.105	0.097	0.026	0.034	0.050	0.024	0.046	0.056	0.037	0.051
*C*_24_	0.096	0.142	0.141	0.111	0.095	0.055	0.055	0.093	0.064	0.115	0.048	0.109	0.127	0.063	0.102	0.086	0.101	0.080
*C*_25_	0.078	0.126	0.132	0.088	0.106	0.044	0.057	0.064	0.116	0.057	0.032	0.052	0.076	0.033	0.048	0.093	0.055	0.073
*C*_31_	0.145	0.160	0.157	0.156	0.147	0.090	0.085	0.108	0.139	0.153	0.048	0.127	0.129	0.067	0.137	0.126	0.140	0.148
*C*_32_	0.131	0.101	0.137	0.148	0.124	0.060	0.055	0.066	0.090	0.112	0.079	0.055	0.120	0.087	0.084	0.140	0.108	0.121
*C*_33_	0.169	0.198	0.193	0.186	0.168	0.085	0.089	0.113	0.135	0.170	0.127	0.149	0.098	0.105	0.144	0.187	0.162	0.164
*C*_34_	0.136	0.173	0.173	0.154	0.151	0.086	0.084	0.094	0.098	0.134	0.103	0.134	0.151	0.049	0.124	0.162	0.149	0.150
*C*_35_	0.052	0.115	0.098	0.088	0.057	0.029	0.032	0.068	0.071	0.069	0.058	0.044	0.068	0.040	0.039	0.116	0.070	0.053
*C*_41_	0.081	0.151	0.152	0.137	0.076	0.045	0.052	0.078	0.090	0.125	0.040	0.057	0.099	0.049	0.094	0.071	0.093	0.096
*C*_42_	0.127	0.165	0.176	0.163	0.118	0.069	0.057	0.071	0.100	0.118	0.083	0.131	0.124	0.094	0.121	0.165	0.076	0.144
*C*_43_	0.128	0.140	0.151	0.106	0.090	0.085	0.055	0.053	0.116	0.142	0.041	0.053	0.092	0.037	0.070	0.138	0.090	0.058

**Table A3:** The total influence matrix of criteria *T*_D_ and sum of influences given/received on dimensions.

Dimensions	*D*_1_	*D*_2_	*D*_3_	*D*_4_	$o_{i}$	$r_{i}$	$\;o_{i}+r_{i}$	$o_{i}-r_{i}$
Economic sustainability (*D*_1_)	0.107	0.077	0.070	0.101	0.356	0.468	0.824	−0.112
Environmental sustainability (*D*_2_)	0.089	0.072	0.048	0.058	0.266	0.325	0.591	−0.059
Social sustainability (*D*_3_)	0.141	0.093	0.095	0.133	0.461	0.291	0.752	0.170
Cultural sustainability (*D*_4_)	0.131	0.084	0.079	0.103	0.397	0.395	0.792	0.001

**Table A4:** Sum of influences given/received on criteria.

Criteria	$o_{i}$	$r_{i}$	$\;o_{i}+r_{i}$	$o_{i}-r_{i}$
Discovering new advantages (*C*_11_)	0.677	0.427	1.104	0.250
Diversification of creative talents (*C*_12_)	0.519	0.583	1.102	−0.064
Increase of symbolic value (*C*_13_)	0.450	0.607	1.057	−0.156
Continuous innovation of products(*C*_14_)	0.474	0.550	1.024	−0.075
Stable financial base (*C*_15_)	0.562	0.516	1.078	0.046
Energy conservation and emission reduction in buildings (*C*_21_)	0.338	0.270	0.608	0.068
Recycling energy (*C*_22_)	0.399	0.256	0.654	0.143
Biodiversity (*C*_23_)	0.334	0.349	0.683	−0.015
High-quality constructed environment (*C*_24_)	0.381	0.458	0.838	−0.077
Unique spatial environment (*C*_25_)	0.338	0.456	0.793	−0.118
Highly effective civic participation mechanism (*C*_31_)	0.508	0.415	0.923	0.093
Widespread interaction and communication (*C*_32_)	0.426	0.509	0.935	−0.083
Conducive public atmosphere to learning (*C*_33_)	0.624	0.567	1.191	0.057
High-quality transmission of information (*C*_34_)	0.562	0.348	0.910	0.213
Sound welfare system (*C*_35_)	0.248	0.528	0.777	−0.280
Amalgamation of diverse cultures (*C*_41_)	0.260	0.374	0.634	−0.114
Self-initiation of cultural activities (*C*_42_)	0.385	0.259	0.643	0.126
Preservation of cultural heritage (*C*_43_)	0.285	0.298	0.583	−0.013

**Table A5:** The un-weighted supermatrix $W_{}^{\alpha }$.

Criteria	*C*_11_	*C*_12_	*C*_13_	*C*_14_	*C*_15_	*C*_21_	*C*_22_	*C*_23_	*C*_24_	*C*_25_	*C*_31_	*C*_32_	*C*_33_	*C*_34_	*C*_35_	*C*_41_	*C*_42_	*C*_43_
*C*_11_	0.111	0.185	0.174	0.171	0.171	0.135	0.208	0.164	0.165	0.147	0.190	0.204	0.185	0.173	0.126	0.136	0.169	0.208
*C*_12_	0.231	0.143	0.254	0.193	0.261	0.157	0.155	0.221	0.243	0.238	0.210	0.158	0.216	0.220	0.281	0.253	0.220	0.228
*C*_13_	0.235	0.229	0.142	0.274	0.239	0.298	0.233	0.294	0.241	0.249	0.205	0.213	0.211	0.219	0.240	0.255	0.235	0.245
*C*_14_	0.224	0.258	0.194	0.129	0.206	0.201	0.212	0.193	0.189	0.166	0.203	0.231	0.204	0.196	0.214	0.230	0.218	0.172
*C*_15_	0.199	0.185	0.235	0.233	0.123	0.210	0.191	0.128	0.162	0.199	0.192	0.193	0.184	0.192	0.139	0.126	0.158	0.147
*C*_21_	0.145	0.134	0.113	0.122	0.151	0.062	0.246	0.156	0.144	0.130	0.157	0.158	0.143	0.174	0.107	0.116	0.167	0.188
*C*_22_	0.174	0.150	0.123	0.146	0.134	0.199	0.070	0.147	0.144	0.169	0.148	0.144	0.151	0.170	0.117	0.133	0.136	0.121
*C*_23_	0.193	0.174	0.169	0.173	0.194	0.234	0.208	0.091	0.244	0.189	0.188	0.172	0.191	0.190	0.254	0.199	0.172	0.116
*C*_24_	0.234	0.263	0.294	0.255	0.280	0.242	0.228	0.314	0.167	0.345	0.241	0.235	0.228	0.197	0.264	0.232	0.240	0.258
*C*_25_	0.255	0.279	0.302	0.305	0.241	0.263	0.247	0.292	0.301	0.167	0.267	0.292	0.287	0.269	0.257	0.320	0.285	0.316
*C*_31_	0.137	0.151	0.143	0.140	0.150	0.124	0.124	0.147	0.107	0.131	0.094	0.186	0.204	0.184	0.232	0.117	0.150	0.140
*C*_32_	0.222	0.210	0.211	0.197	0.206	0.176	0.171	0.189	0.243	0.215	0.250	0.130	0.239	0.238	0.178	0.169	0.237	0.180
*C*_33_	0.272	0.277	0.286	0.301	0.241	0.252	0.254	0.278	0.284	0.315	0.255	0.281	0.158	0.269	0.274	0.292	0.225	0.314
*C*_34_	0.151	0.124	0.172	0.180	0.135	0.146	0.136	0.131	0.139	0.138	0.132	0.205	0.168	0.088	0.160	0.145	0.170	0.127
*C*_35_	0.218	0.237	0.188	0.183	0.269	0.301	0.314	0.256	0.227	0.201	0.269	0.198	0.231	0.221	0.156	0.277	0.218	0.238
*C*_41_	0.431	0.415	0.409	0.328	0.342	0.341	0.368	0.388	0.322	0.421	0.304	0.381	0.365	0.353	0.488	0.272	0.429	0.482
*C*_42_	0.234	0.305	0.337	0.277	0.339	0.277	0.307	0.260	0.377	0.247	0.338	0.292	0.316	0.323	0.292	0.358	0.196	0.315
*C*_43_	0.336	0.279	0.254	0.395	0.319	0.382	0.324	0.352	0.301	0.332	0.358	0.328	0.320	0.325	0.220	0.370	0.374	0.203

**Table A6:** The weighted supermatrix W.

Criteria	*C*_11_	*C*_12_	*C*_13_	*C*_14_	*C*_15_	*C*_21_	*C*_22_	*C*_23_	*C*_24_	*C*_25_	*C*_31_	*C*_32_	*C*_33_	*C*_34_	*C*_35_	*C*_41_	*C*_42_	*C*_43_
*C*_11_	0.033	0.056	0.053	0.052	0.052	0.045	0.070	0.055	0.055	0.049	0.058	0.062	0.056	0.053	0.038	0.045	0.056	0.068
*C*_12_	0.070	0.043	0.077	0.058	0.079	0.052	0.052	0.074	0.081	0.080	0.064	0.048	0.066	0.067	0.086	0.083	0.073	0.075
*C*_13_	0.071	0.069	0.043	0.083	0.072	0.100	0.078	0.098	0.081	0.083	0.063	0.065	0.064	0.067	0.073	0.084	0.077	0.081
*C*_14_	0.068	0.078	0.058	0.039	0.062	0.067	0.071	0.065	0.063	0.056	0.062	0.071	0.062	0.060	0.065	0.076	0.072	0.057
*C*_15_	0.060	0.056	0.071	0.070	0.037	0.070	0.064	0.043	0.054	0.067	0.059	0.059	0.056	0.059	0.042	0.042	0.052	0.048
*C*_21_	0.031	0.029	0.024	0.026	0.033	0.017	0.066	0.042	0.039	0.035	0.031	0.032	0.029	0.035	0.021	0.025	0.035	0.040
*C*_22_	0.038	0.033	0.027	0.032	0.029	0.053	0.019	0.039	0.039	0.045	0.030	0.029	0.030	0.034	0.024	0.028	0.029	0.026
*C*_23_	0.042	0.038	0.037	0.037	0.042	0.063	0.056	0.025	0.065	0.051	0.038	0.034	0.038	0.038	0.051	0.042	0.036	0.025
*C*_24_	0.051	0.057	0.064	0.055	0.061	0.065	0.061	0.084	0.045	0.093	0.048	0.047	0.046	0.040	0.053	0.049	0.051	0.055
*C*_25_	0.055	0.060	0.065	0.066	0.052	0.071	0.067	0.078	0.081	0.045	0.054	0.059	0.058	0.054	0.052	0.068	0.060	0.067
*C*_31_	0.027	0.030	0.028	0.027	0.030	0.022	0.022	0.026	0.019	0.024	0.019	0.038	0.042	0.038	0.048	0.023	0.030	0.028
*C*_32_	0.044	0.041	0.041	0.039	0.040	0.032	0.031	0.034	0.044	0.039	0.051	0.027	0.049	0.049	0.037	0.034	0.047	0.036
*C*_33_	0.053	0.055	0.056	0.059	0.047	0.045	0.046	0.050	0.051	0.057	0.052	0.058	0.032	0.055	0.056	0.058	0.045	0.062
*C*_34_	0.030	0.024	0.034	0.035	0.026	0.026	0.025	0.024	0.025	0.025	0.027	0.042	0.035	0.018	0.033	0.029	0.034	0.025
*C*_35_	0.043	0.047	0.037	0.036	0.053	0.054	0.056	0.046	0.041	0.036	0.055	0.041	0.048	0.045	0.032	0.055	0.043	0.047
*C*_41_	0.123	0.118	0.117	0.093	0.098	0.074	0.080	0.084	0.070	0.091	0.088	0.110	0.105	0.102	0.141	0.071	0.112	0.126
*C*_42_	0.067	0.087	0.096	0.079	0.096	0.060	0.067	0.056	0.082	0.053	0.097	0.084	0.091	0.093	0.084	0.093	0.051	0.082
*C*_43_	0.096	0.080	0.072	0.112	0.091	0.083	0.070	0.076	0.065	0.072	0.103	0.095	0.092	0.094	0.064	0.096	0.097	0.053

**Table A7:** The influential weights of DANP when $lim_{z\to \infty }{\left(W\right)}^{z}$.

Criteria	*C*_11_	*C*_12_	*C*_13_	*C*_14_	*C*_15_	*C*_21_	*C*_22_	*C*_23_	*C*_24_	*C*_25_	*C*_31_	*C*_32_	*C*_33_	*C*_34_	*C*_35_	*C*_41_	*C*_42_	*C*_43_
IWs	0.0529	0.0700	0.0749	0.0640	0.0552	0.0320	0.0318	0.0411	0.0569	0.0622	0.0286	0.0396	0.0529	0.0291	0.0449	0.1011	0.0793	0.0835
